# Species diversity and phylogeography of *Cornus kousa* (Asian dogwood) captured by genomic and genic microsatellites

**DOI:** 10.1002/ece3.6537

**Published:** 2020-07-11

**Authors:** Marcin Nowicki, Logan C. Houston, Sarah L. Boggess, Anthony S. Aiello, Miriam Payá‐Milans, Margaret E. Staton, Mitsuhiro Hayashida, Masahiro Yamanaka, Shigetoshi Eda, Robert N. Trigiano

**Affiliations:** ^1^ Department of Entomology and Plant Pathology The University of Tennessee Knoxville TN USA; ^2^ Morris Arboretum University of Pennsylvania Philadelphia PA USA; ^3^ Faculty of Agriculture Yamagata University Tsuruoka Japan; ^4^ Department of Pharmaceytical Sciences International University of Health and Welfare Ohtawara Japan; ^5^ Department of Forestry, Wildlife and Fisheries Center for Wildlife Health ORU Knoxville TN USA; ^6^ Department of Microbiology Center for Wildlife Health ORU Knoxville TN USA; ^7^ Present address: Centro de Biotecnología y Genómica de Plantas UPM‐INIA Madrid Spain

**Keywords:** dogwoods, evolutionary history, plant species diversity, population genetics

## Abstract

*Cornus kousa* (Asian dogwood), an East Asia native tree, is the most economically important species of the dogwood genus, owing to its desirable horticultural traits and ability to hybridize with North America‐native dogwoods. To assess the species genetic diversity and to better inform the ongoing and future breeding efforts, we assembled an herbarium and arboretum collection of 131 noncultivated *C. kousa* specimens. Genotyping and capillary electrophoresis analyses of our *C. kousa* collection with the newly developed genic and published nuclear genomic microsatellites permitted assessment of genetic diversity and evolutionary history of the species. Regardless of the microsatellite type used, the study yielded generally similar insights into the *C. kousa* diversity with subtle differences deriving from and underlining the marker used. The accrued evidence pointed to the species distinct genetic pools related to the plant country of origin. This can be helpful in the development of the commercial cultivars for this important ornamental crop with increased pyramided utility traits. Analyses of the *C. kousa* evolutionary history using the accrued genotyping datasets pointed to an unsampled ancestor population, possibly now extinct, as per the phylogeography of the region. To our knowledge, there are few studies utilizing the same gDNA collection to compare performance of genomic and genic microsatellites. This is the first detailed report on *C. kousa* species diversity and evolutionary history inference.

## Graphical abstract

*Cornus kousa* (Asian dogwood) is the most economically important species of the dogwood genus, owing to its desirable ornamental traits and ability to hybridize with North America‐native flowering dogwoods. To assess the species genetic diversity and to better inform the ongoing and future breeding efforts, we genotyped a collection of noncultivated *C. kousa* from US herbaria and arboreta samples using genomic an genic microsatellites. The data pointed to the species distinct genetic pools related to the tree country of origin, and informed the species phylogeographic history, which can help future breeding efforts.

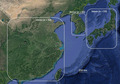

## 1 INTRODUCTION

Asian dogwood (*Cornus kousa* F. Buerger ex Hance), a small‐ to medium‐sized deciduous tree, is a counterpart to the North America‐native *C. florida* L. (Cappiello & Shadow, 2005; Wadl, Wang, Scheffler, Wang, Scheffler, Rinehart, & Trigiano, 2008). Because of the popularity of commercial cultivars in the nursery and landscape industries, both species generate the most economic value from among about 70 species currently recognized in the genus *Cornus* L. (http://www.theplantlist.org/; Cappiello & Shadow, 2005; Nowicki et al., 2018; Wadl, Windham, Windham, Evans, & Trigiano, 2014). The annual sales in the United States of America (USA) alone amount to about 3 million trees (Nowicki et al., 2018; USDA‐NASS, 2015; Wadl, Windham, et al., 2014). Sales exceeding $30 M underscore the market demand for about 150 commercial cultivars of either species (Cappiello & Shadow, 2005; Nowicki et al., 2018; Wadl, Windham, et al., 2014). In particular, past and ongoing hybrid breeding programs between *C. kousa* with several US‐native dogwood species have been pursued (Cappiello & Shadow, 2005; Mattera, 2016; Mattera, Molnar, & Struwe, 2015; Molnar, Muehlbauer, Muehlbauer, Wadl, & Capik, 2017; Shearer & Ranney, 2013a, 2013b). Those aimed to utilize many desirable horticultural traits of *C. kousa*, such as pathogen resistance, abiotic stress tolerance, and an extended flowering period (Cappiello & Shadow, 2005; Li et al.., 2007; Thurn, Lamb, & Eshenaur, 2018). Genomic resources for this diploid species are scarce, but the nuclear genome size (1C = 1.92 pg) and the base chromosome number of 1C = 11 are known (Dermen, 1932; Goldblatt, 1978; Shearer & Ranney, 2013a, 2013b). To better inform the existing and future breeding programs of dogwoods, knowledge of the *C. kousa* genetic diversity is needed, as previous research indicated that several commercially available cultivars were either very closely related or indeed clones of the same plants (Molnar et al., 2017; Trigiano, Ament, Windham, & Moulton, 2004).

Microsatellites (short‐sequence repeats; short tandem repeats; SSRs) are considered a valuable tool for inferring species genetic diversity and population structure (Ellis & Burke, 2007; Pritchard, Stephens, & Donnelly, 2000). Genomic sequences (nuclear), transcribed genomic sequences (transcriptomic; genic), and cytoplasmic genomic sequences (plastidial/mitochondrial) can all be searched for SSRs (Ellis & Burke, 2007; Nowicki et al., 2018; Rubinsztein et al., 1995). Owing to the differences in the character and organization of the originating genomic sequence, the resultant SSRs tend to show minor differences in the genetic distances captured and therefore might be preferentially used in projects varying in scope (Bhargava & Fuentes, 2010; Nowicki, Zhao, et al., 2019; Rubinsztein et al., 1995). The relatively high mutation rates of SSRs render them an especially attractive tool for the analyses of population and higher levels of organism organization (Bhargava & Fuentes, 2010; Nowicki, Schilling, et al., 2019; Raquin et al., 2008; Rubinsztein et al., 1995). SSRs have been used as a functional tool in *C. florida* genetic diversity studies (Hadziabdic, 2010; Hadziabdic et al., 2010, 2012) and were developed for several other dogwood species (Keir, Bemmels, & Aitken, 2011; Mattera et al., 2015; Wadl, Szyp‐Borowska, et al., 2014; Wadl et al., 2010), including *C. kousa* (Wadl, Wang, Scheffler, et al., 2008; Wadl, Wang, Trigiano, et al., 2008).

The use of the SSRs exceeds the conventional snapshot analyses of species diversity, with the currently available tools and algorithms offering insights into their evolutionary histories as well (Kim et al., 2019; Zhang et al., 2019). For instance, SSR datasets can help make powerful evolutionary inferences regarding how the species history was shaped to present state. It is achieved by using a combination of the available geological resources that illustrate topology and climate changes, fossil records that evidence species occurrence and evolution, and species biology and ecology that inform the mode of reproduction, natural threats, or dispersal (Jeon, 2018; Riser, Emel, & Roalson, 2019; Zhang et al., 2019). Such studies were also performed for two North America‐native dogwoods *C. florida* (Call, 2012; Call et al., 2015) and *C. nuttallii* Audubon ex Torr. & A.Gray (Keir, 2008; Keir et al., 2011), leading to species‐level conclusions about their current genetic diversity and informing future conservation efforts.

Recognizing the economic potential of the Asian dogwood, we aimed to assess its currently unknown genetic diversity. To reach this goal, we developed the genic SSRs (eSSRs) and utilized the available genomic SSRs (gSSRs; Wadl et al., 2010; Wadl, Wang, Scheffler, et al., 2008) on the available and historical specimens of native, noncultivated samples of *C. kousa*. Doing so allowed us to accrue information on how the species genetic diversity was captured by either SSR type, whereas the exhaustive analytical pipeline allowed inferences about the species phylogeographic history.

## 2 MATERIALS AND METHODS

### 2.1 Plant materials and gDNA extraction

**FIGURE 1:**
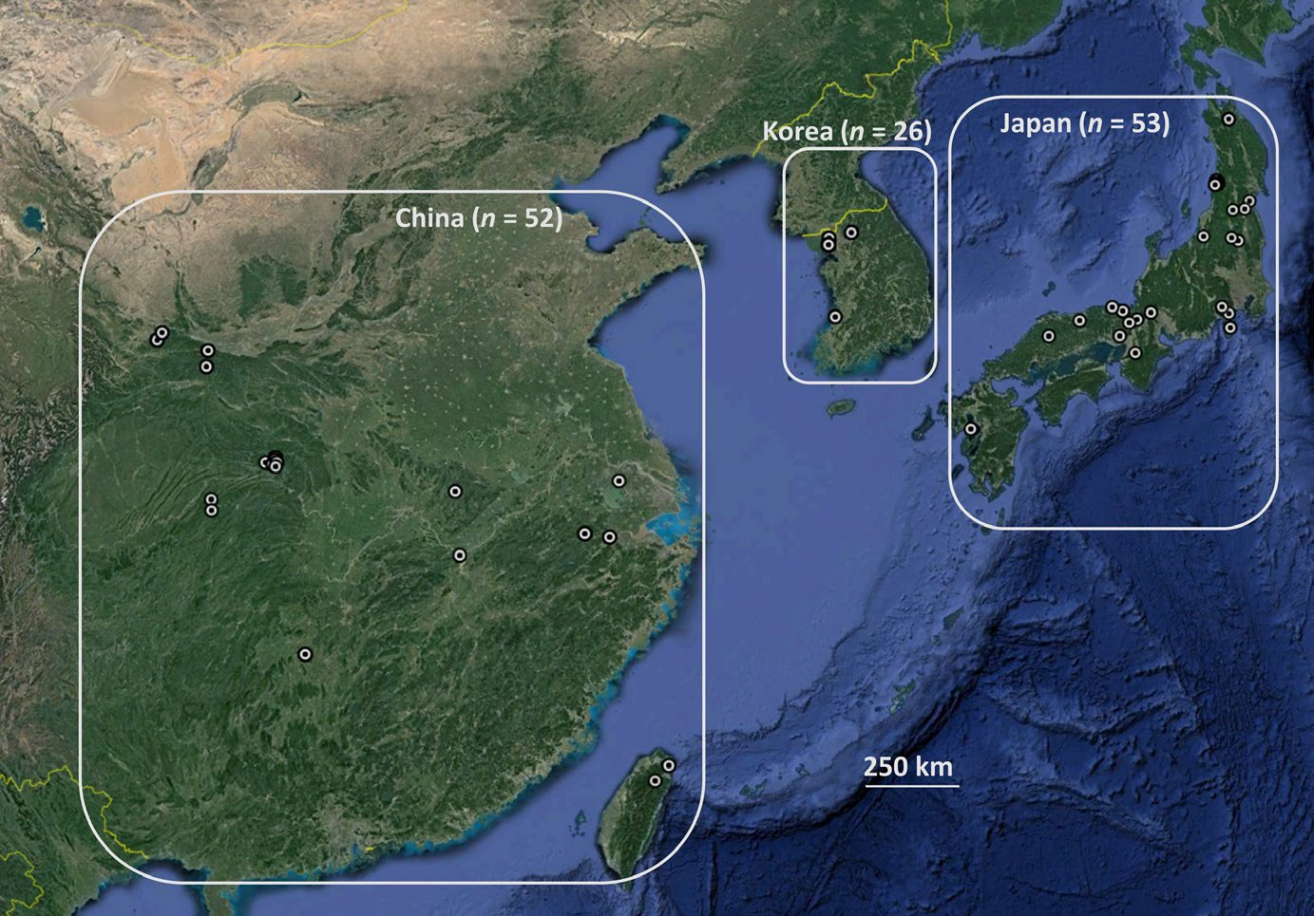
Map of the origin of the *Cornus kousa* individuals sampled for the study. Eye altitude: 4,010 km above sea level. Each subpopulation is marked with the country of origin, and their respective counts are listed out. Individuals overlap in several locations due to the scale of the presentation. Also, see Table 1 and Appendix S1. The region maps were visualized using Google Earth Pro version 7.3.2.5491. The GPS coordinates of our *C. kousa* collection are shown as whitish hollow circles. The scale line indicates a ground‐level distance of 250 km

Samples of noncultivated *C. kousa* were collected from living plants and herbarium specimens from the species native range (China: *n* = 52; Japan: *n* = 53; Korea: *n* = 26; described in detail in Table 1; Appendix S1; Figure 1). We sampled leaf and/or flower buds from wild plants living in China and Japan and those grown from Asian wild‐collected seeds/seedlings/clones growing in the United States (Morton Arboretum, Lisle, IL; U.S. National Arboretum, Beltsville, MD; and Morris Arboretum of the University of Pennsylvania, Philadelphia, PA; collected 1984 through 2018). No special permissions were required for those collections of *C. kousa* as the materials tested were the wild‐growing, noncultivated *C. kousa* trees and the species is not classified as endangered or protected. Historical specimens of the wild‐growing *C. kousa* of Asian origin were submitted for limited destructive sampling upon our request from the Carnegie Museum of Natural History Herbarium (Pittsburgh, PA) and Ray J. Davis Herbarium, Idaho State University (Pocatello, ID). The specimens were collected from 1953 to 1992 and were dated as per their collection year. Specimens were grouped into meta‐populations per their country of origin, with the land contiguity in mind.

**TABLE 1:** Population genetics indices for subpopulations of *Cornus kousa* generated with the combined short‐sequence repeat dataset (egSSR)

Population	*N* ^a^	%amp	*N*_P_	*P*_A_	*N*_E_	*H*	*λ*	*I*_A_	${\bar{r}}_{d}$	*A*_R_	*µH_E_*	*H*_O_	*F*_I_	*I*
China	51	93.5	252	49	3.96	3.93	0.98	1.83^***^	0.056^***^	5.95	0.63	0.21	0.664^***^	1.34
Japan	46	94.0	235	43	4.13	3.83	0.98	1.65^***^	0.052^***^	5.77	0.65	0.22	0.660^***^	1.37
Korea	26	98.2	164	12	2.95	3.26	0.96	1.74^***^	0.055^***^	4.41	0.51	0.21	0.599^***^	1.01
Overall	123	94.7	313		4.39	4.81	0.99	1.37^***^	0.042^***^	6.24	0.65	0.22	0.671^***^	1.24

^a^ *N*: number of individuals tested; %amp: % of samples amplified from a total from the *N* column across all 37 SSRs; *N*_P_: number of alleles detected per population; *P*_A_: number of private alleles per population; *N*_E_ = number of effective alleles (Nielsen, Tarpy, & Reeve, 2003); *H*: Shannon–Weiner Index of MLG diversity (Shannon, 2001; increases together with both richness and evenness of the species present); *λ*: Simpson's index (Simpson, 1949); *I*_A_ Index of Association assesses whether the loci are linked (Kamvar et al., 2014, 2015); ${\bar{r}}_{d}$: Index of Association that accounts for the number of loci sampled and is thus less biased (Kamvar et al., 2014, 2015); *A*_R_: allelic richness (expected number of alleles among 36 gene copies; Korea: CK040); *µH*_E_: Nei's unbiased gene diversity corrected for sample size (Nei, 1978); *H*_O_ = observed heterozygosity; *F*_I_ = Fixation index—individual inbreeding coefficient. *I*: Shannon's information index (Shannon, 2001). Significance was assessed by 10,000 permutations of the dataset. ^***^ *p* < .001 at 10,000 permutations.

Total DNA (gDNA) was extracted from the samples of the living specimens using the DNeasy Plant Mini Kit (Qiagen Inc., Germantown, MD, USA) with minor protocol modifications (Nowicki et al., 2018). A collection of 15 gDNA samples from wild specimens from China described in a previous study was also included (Nowicki et al., 2018). Twenty‐one samples from noncultivated Japanese accessions were collected, and gDNA was extracted using the MagExtractor‐Plant Genome Kit (Toyobo Co. Ltd) according to the manufacturer's protocol. gDNA from the historical herbarium specimens was isolated using the E.Z.N.A. Plant DNA Kit (Omega Bio‐tek) according to the manufacturer's protocol. Isolated gDNA was evaluated for integrity by electrophoresis in 2% agarose gels stained with ethidium bromide for DNA visualization under the UV light. Purity and concentration were assessed using NanoDrop ND‐1000 UV/Vis (Fisher Scientific).

### 2.2 Molecular markers used for the study (gSSR, eSSR development)

Genomic SSRs (gSSRs) used in this research project were developed in previous studies (Wadl, Wang, Scheffler, et al., 2008; Wadl, Wang, Trigiano, et al., 2008). They were successfully used in other projects (Wadl et al., 2009, 2010) and in developing the new cultivars (Molnar et al., 2017; Wadl, Windham, et al., 2014). We tested 20 gSSRs on the entire collection of *C. kousa* gDNA in this study (Table 2; Appendix S1).

**TABLE 2:** Population genetics indices for the SSR loci of the *Cornus kousa* dataset generated with the genomic short‐sequence repeat markers (CK#) and genic SSRs (Ckest#)

SSR locus	*N*_L_ ^a^	*N*_E_	*A*_R_	*µH_E_*	*H_O_*	*F*_IS_	*F*_ST_	*R*_IS_	*R*_ST_	*D*_est_	*G*	E.5	*Nm*
Ckest01	8	2.7	6.4	0.63	0.11	0.79^***^	0.19^***^	0.63^***^	0.13^**^	0.25^***^	2.72	0.56	1.36
Ckest02	6	2.8	4.5	0.64	0.00	1.00^***^	0.07^**^	1.00^***^	0.09^*^	0.09^*^	2.76	0.77	4.26
Ckest06	9	3.5	5.7	0.72	0.06	0.88^***^	0.40^***^	0.80^***^	0.40^***^	0.62^***^	3.50	0.75	0.49
Ckest09	7	4.2	5.2	0.76	0.08	0.89^***^	0.07^**^	0.87^***^	0.13^**^	0.18^**^	4.18	0.89	3.86
Ckest17	5	2.0	3.3	0.50	0.15	0.66^***^	0.14^***^	0.32^**^	0.07^*^	0.14^***^	2.01	0.74	1.68
Ckest18	11	2.7	7.0	0.64	0.25	0.60^***^	0.03^ns^	0.72^***^	0.07^*^	0.09^**^	2.75	0.53	5.39
Ckest22	5	3.7	4.3	0.73	0.01	0.99^***^	0.08^**^	1.00^***^	0.10^**^	0.21^**^	3.66	0.91	2.80
Ckest23	6	2.6	5.0	0.62	0.14	0.77^***^	0.03^ns^	0.84^***^	0.00^ns^	0.07^*^	2.62	0.69	4.93
Ckest24	10	4.1	6.2	0.75	0.02	0.98^***^	0.07^**^	0.91^***^	0.05^ns^	0.24^***^	4.05	0.77	3.05
Ckest27	9	5.3	6.9	0.81	0.21	0.72^***^	0.12^***^	0.76^***^	0.23^***^	0.40^***^	5.20	0.83	2.29
Ckest28	6	2.6	4.2	0.61	0.00	1.00^***^	0.25^***^	1.00^***^	0.39^***^	0.29^***^	2.55	0.77	1.10
Ckest33	11	4.0	6.2	0.75	0.08	0.89^***^	0.09^***^	0.50^**^	0.04^ns^	0.22^***^	3.96	0.76	2.97
Ckest41	4	1.5	3.0	0.32	0.06	0.82^***^	0.01^ns^	0.80^***^	0.03^ns^	0.01^ns^	1.47	0.57	9.48
Ckest46	4	2.2	3.6	0.54	0.07	0.86^***^	0.06^*^	0.75^***^	0.03^ns^	0.06^*^	2.17	0.73	4.12
Ckest47	3	1.0	1.7	0.04	0.02	0.38^ns^	0.02^ns^	−0.04^ns^	0.05^*^	0.00^*^	1.04	0.35	8.38
Ckest48	5	1.4	3.5	0.27	0.03	0.91^***^	0.01^ns^	0.78^***^	0.00^ns^	0.00^ns^	1.37	0.46	10.95
Ckest49	5	1.2	2.8	0.15	0.01	0.94^***^	0.03^ns^	1.00^***^	0.07^*^	0.01^*^	1.17	0.40	4.95
CK005	26	11.5	15.3	0.91	0.34	0.61^***^	0.07^***^	0.86^***^	−0.02^ns^	0.54^***^	10.99	0.67	3.93
CK015	15	9.5	11.5	0.89	0.47	0.45^***^	0.08^***^	0.44^***^	0.20^***^	0.46^***^	9.21	0.82	3.81
CK016	3	1.8	2.5	0.44	0.00	1.00^***^	0.11^**^	1.00^***^	0.05^ns^	0.07^**^	1.78	0.79	2.61
CK029	7	3.2	4.9	0.68	0.04	0.92^***^	0.22^***^	0.94^***^	0.08^*^	0.41^***^	3.13	0.78	1.07
CK031	7	3.0	5.6	0.67	0.25	0.58^***^	0.14^***^	0.27^**^	0.35^***^	0.21^***^	3.05	0.68	2.15
CK040	14	6.6	10.5	0.85	0.18	0.78^***^	0.03^*^	0.74^***^	−0.02^ns^	0.15^*^	6.41	0.69	5.51
CK043	11	4.4	6.4	0.77	0.83	−0.15^**^	0.10^***^	−0.68^***^	0.07^***^	0.32^***^	4.32	0.78	2.86
CK047	7	4.9	5.3	0.80	0.11	0.85^***^	0.05^**^	0.72^***^	0.05^ns^	0.17^*^	4.82	0.94	4.57
CK058	19	8.0	10.3	0.87	0.72	0.15^***^	0.05^***^	−0.11^ns^	0.08^**^	0.28^***^	7.73	0.77	5.41
CK065	16	7.7	10.7	0.87	0.43	0.49^***^	0.06^***^	0.53^***^	0.06^*^	0.34^***^	7.46	0.74	3.57
CK066	16	10.7	12.5	0.91	0.54	0.39^***^	0.04^***^	0.51^***^	−0.01^ns^	0.32^***^	10.32	0.83	5.43
CK068	12	9.7	10.5	0.90	0.36	0.57^***^	0.07^***^	0.62^***^	0.23^***^	0.44^***^	9.41	0.91	3.53
CK070	6	2.5	3.7	0.60	0.01	0.99^***^	0.07^*^	0.82^***^	0.10^**^	0.11^**^	2.53	0.78	3.41
CK071	10	6.5	7.7	0.85	0.14	0.83^***^	0.08^***^	0.87^***^	0.23^***^	0.36^***^	6.42	0.88	3.15
CK072	6	2.0	3.6	0.50	0.23	0.51^***^	0.07^**^	0.19^*^	0.02^ns^	0.08^**^	2.01	0.68	3.04
CK076	3	1.9	3.0	0.48	0.00	1.00^***^	0.00^ns^	1.00^***^	0.00^ns^	0.00^ns^	1.91	0.70	16.39
CK089	4	2.1	2.7	0.53	0.98	−0.88^***^	0.00^ns^	−0.96^***^	0.00^ns^	0.00^ns^	2.10	0.89	114.10
CK092	17	10.3	12.2	0.90	0.62	0.30^***^	0.05^***^	0.18^ns^	0.09^**^	0.41^***^	9.92	0.82	4.36
Overall	8.9	4.4	6.2	0.65	0.22	0.65^***^	0.09^***^	0.48^***^	0.10^***^	0.16^***^	4.31	0.73	7.46

^a^ *N*_L_: number of alleles detected; *N*_E_: estimated number of effective alleles; *A*_R_: allelic richness (expected number of alleles among 36 gene copies); *H*_O_: observed heterozygosity (frequency of heterozygous individuals per locus averaged over number of sampled loci); *µH*_E_: expected heterozygosity (Nei's unbiased gene diversity; Nei, 1978 ); Wright's fixation indices *F*_ST_: a measure of subpopulation genetic structure; *F*_IS_: a measure of deviations from Hardy–Weinberg equilibrium with regard to homozygote excess if >0 or deficiency if <0); *R*_ST_ and *R*_IT_: *F*_ST_ and *F*_IS_ analogues based on allele size (number of repeats; Slatkin, 1995; estimated as Michalakis & Excoffier, 1996); *D*_est_: Jost's estimate of differentiation (Jost, 2008); *G*: Stoddard and Taylor index (Stoddard & Taylor 1988); E.5: evenness (relative abundance of allele species); *Nm*: gene flow (*Nm* = ¼ × [(1/*F*_ST_) − 1]). Significance was assessed by 10,000 permutations of the dataset. ^***^ *p* < .001; ^**^ *p* < .01; ^*^ *p* < .05; ^ns^ *p *> .05 at 10,000 permutations.

The genic SSRs (eSSRs) were developed based on the available *C. kousa* transcriptome (Yu et al., 2017). Transcriptome completeness was assessed with the Benchmarking Universal Single‐Copy Ortholog (BUSCO) analysis using the Embryophyta database (Simao, Waterhouse, Ioannidis, Kriventseva, & Zdobnov, 2015). Low‐complexity regions of the *C. kousa* transcriptome were masked out with Dustmasker (Morgulis, Gertz, Schaffer, & Agarwala, 2006) with a level of 1 prior to finding SSRs within the assembled transcriptome. This study used a custom perl script to identify the eSSRs within the analyzed transcriptome and generate the primers using Primer3 (Untergasser et al., 2012). The script searched for the perfect repeats of di‐, tri‐, and tetra‐nucleotide motifs, repeated between (min.) 6, 6, or 4 and (max.) 20 times, respectively, looking only for primers that together with the SSR amplified a fragment ranged in size between 100 and 500 bp.

### 2.3 Genotyping conditions and primer screening

Twenty gSSRs from previous studies (Wadl et al., 2009, 2010; Wadl, Wang, Scheffler, et al., 2008; Wadl, Wang, Trigiano, et al., 2008) were screened across the entire gDNA collection using PCR, and 50 eSSRs (20 di‐, 20 tri‐, and 10 tetra‐repeats) were prescreened on a single gDNA sample (“Morris43”; Appendix S1). Based on this preliminary data, we then selected 31 eSSRs to screen the entire gDNA collection of *C. kousa*. The reaction of 10 µl contained 4 µl of 2 × AccuStart II PCR SuperMix (QuantaBio), 0.5 µM of each primer (Integrated DNA Technologies, Inc.), and 4 ng of gDNA. The touch‐down thermal profile for amplification of both types of SSRs was as follows: initial denaturation at 94°C for 3 min; 10 touch‐down cycles of 94°C for 40 s, 63°C of 40 s (−0.5°C/cycle), and 72°C for 30 s; followed by 35 cycles of 94°C for 40 s, 58°C for 40 s, and 72°C for 30 s; and final extension at 72°C for 4 min. The PCR products were visualized using the QIAxcel Advanced electrophoresis capillary electrophoresis (Qiagen) and analyzed using a 25‐ to 500‐bp DNA size marker and an internal 15/600‐bp alignment marker. We screened the results of genotyping with 20 gSSRs and 31 eSSRs for specificity (including agreement with the previous studies and the product sizes expected by the transcriptome, respectively), amplification of more than 70% of *C. kousa* gDNA collection, and productivity. Based on these criteria, the results of amplification with 18 gSSRs and 17 eSSRs (four di‐, eight tri‐, and five tetra‐repeats; Table 2; Appendix S1) were selected and used for the subsequent analyses. Two additional genotyping attempts were carried out on the samples with missing allele(s) to confirm no amplification of these putative alleles was possible. Samples with missing data at >50% loci (eSSR or gSSR) were discarded from the subsequent analyses after all three attempts at genotyping failed.

### 2.4 Population genetics of *C. kousa*

The genotyped datasets for the gSSRs and eSSRs were binned using FlexiBin, an MS Excel macro (Amos et al., 2007). The binned datasets underwent, together and separately (Appendix S1), an array of population genetic analyses. In 124 gDNA samples, 17 eSSRs (henceforth, eSSR dataset) were amplified. In 131 gDNA samples, 18 gSSRs were amplified (henceforth, gSSR dataset). The combined dataset included 123 gDNA samples of *C. kousa* with information for 35 SSRs (henceforth, egSSR dataset). Clonal correction of those datasets revealed that no identical multilocus genotypes (MLGs) were present in any of the datasets. Datasets were then normalized per each given SSR motif length, to reflect number of repeats rather than PCR allele size (Hardy & Vekemans, 2015; Hey et al., 2018). Hierarchical fixation and differentiation indices (*F*_ST_, *F*_IS_, *R*_ST_, *R*_IS_, and *D*_est_, respectively; Bird, Karl, Smouse, & Toonen, 2011; Jost et al., 2018; Slatkin, 1995) were estimated using the following analytical packages: *poppr* version 2.8.1 (Kamvar, Brooks, & Grunwald, 2015; Kamvar, Tab ima, & Grünwald, 2014) and *hierfstat* version 0.04‐22 (Goudet, 2005; Goudet, Raymond, de Meeus, & Rousset, 1996) in R version 3.4.3 (R Core Team, 2017), and SPAGeDi version 1.5 (Hardy & Vekemans, 2015). Significance of those indices was calculated by 10,000 permutes of the gene copies among individuals within populations, or individuals among all populations, respectively. Population structure of the *C. kousa* genotyping dataset was analyzed using the Bayesian method of structure version 2.3.4 (Pritchard et al., 2000). To infer the genetic clustering among the *C. kousa* individuals, we implemented 30 independent Monte Carlo Markov chains (MCMCs) with 250,000 generations of burn‐in followed by 750,000 of the actual run for each *K* value (1–5). structure results were analyzed with PopHelper version 1.0.10 utilizing the Evanno method (Evanno, Regnaut, & Goudet, 2005; Francis, 2017). Analysis of the factors driving the *C. kousa* population structure by iterative independent removal of the predefined populations or the inferred clusters was carried out using ObStruct (Gayevskiy, Klaere, Knight, & Goddard, 2014). Mantel and partial Mantel tests, that are mathematical tools to compare data matrices, were performed in R using the package MASS version 7.3‐50 (Venables & Ripley, 2002). Mantel correlogram tests were performed in R using the packages ade4 version 1.7‐13 and vegan version 2.5‐3 (Dray & Dufour, 2007; Oksanen et al., 2013). Both analyses used 1,000 permutations of the datasets. Analysis of the molecular variance (AMOVA) was performed in R using the package *poppr*, and the resulting Φ indices of variance partitioning are reported as [%] values, after 1,000 permutations. Discriminant analysis of principal component (DAPC) was performed in R using the package *adegenet* version 2.1.1 (Jombart, 2008; Jombart et al., 2018).

### 2.5 Demographic and evolutionary scenario inferences

Assessment of recent bottleneck or expansion of *C. kousa* was carried out using the program BOTTLENECK version 1.2.02 (Cornuet & Luikart, 1996). The following mutation models were screened: stepwise‐mutation model (S.M.M.) and two‐phase mutational model (T.P.M.; 30% S.M.M.). Significance of this test under either of these models was evaluated by Wilcoxon's sign‐ranked tests with 10,000 simulations (Cornuet & Luikart, 1996; Piry, Luikart, & Cornuet, 1999).

Inferences on the history of *C. kousa* were made with the approximate Bayesian computation approach using the program DIYABC version 2.1 (Cornuet et al., 2014). In our analyses, we focused on the putative geographic origin based on the sampled subpopulations of *C. kousa*. Because the sampling years in our collection spanned about 10 generations (Molnar et al., 2017; Schmidt, 1995), which likely would not make a difference in the inferences reaching thousands of generations back (as confirmed with the partial Mantel test results), we analyzed three subpopulations, as per the sample country of origin (“China,” “Japan,” and “Korea”). Furthermore, this grouping was also supported by the DAPC analyses. Limiting the number of populations with generally shared histories reduces the complexity of the evaluated scenarios and is frequently applied in this method (Cornuet et al., 2014; Jeon, 2018; Kim et al., 2019; Zhang et al., 2019). We undertook a two‐step approach, and in the initial round tested the dataset for 35 possible scenarios that considered sequential divergence, admixture, population size variations, or a “ghost” (unsampled) originating population (Appendix S2). This generated a reference table over 1 M permutations of the dataset per each scenario tested and assumed a generalized S.M.M. with a mean mutation rate of 5 × 10^–4^ (ranging from 10^–6^ to 10^–3^ mutations per generation per locus) and a uniform prior distribution. Similarities between the real and simulated datasets were assessed per each regarded scenario as instructed in the DIYABC manual using the summary statistics both within and between populations. Summary statistics for single populations included the mean number of alleles, mean genetic diversity, and mean size variance. Summary statistics for pairs of populations included pairwise *F*_ST_, classification index, and (*δμ*)^2^ distance for pairs of populations. Principal component analysis (PCA) pre‐evaluation step was also performed to ensure that at least one combination of scenarios and priors produced simulated datasets close enough to the observed dataset. The relative posterior probabilities of the scenarios undergoing comparison were inferred by logistic regression on the 1% of simulated datasets closest to the observed dataset (Cornuet, Ravigné, & Estoup, 2010). Because of the lack of bottleneck evidence for the dataset, only the top‐two simple split scenarios with the highest posterior probabilities from the initial DIYABC round were compared in the separate subsequent analyses. A model with the highest posterior probability and for which the 95% confidence interval (CI) did not overlap with the CIs of the other model was considered the best model. For each dataset, a model‐checking algorithm was also applied to evaluate the goodness of fit of the chosen best scenario using PCA, in which the observations were the simulated datasets and the variables were all the summary statistics listed out above. As per the DIYABC manual, the “confidence in scenario choice” method was applied to estimate the proportion of type 1 and type 2 errors based on 1,000 pseudo‐observed datasets. Small type 2 errors provide good confidence in the selected scenario even if the type 1 errors are large.

## 3 RESULTS

### 3.1 Development of the *C. kousa* genic eSSR markers

BUSCO analysis of the *C. kousa* transcriptome of Yu et al. (2017) determined that out of 1,440 Embryophyta BUSCO‐associated transcripts, 1,180 were found. Of those, in total 1,044 were deemed “complete,” with 928 having a single copy and 116 having duplicates, and 136 were identified as fragmented.

In total, 18,694 SSRs were identified in the 16,230 sequences assembled. Of those, 1,306 SSRs were compound and discarded from further analyses. The remaining 17,388 SSRs resulted in 8,858 SSRs with primers. All 12 unique (i.e., redundantly iterated) dinucleotide‐motif repeat SSRs were found with varying frequencies in the *C. kousa* transcriptome. Of the possible 60 unique trinucleotide‐motif repeats, only the [GTA]*_n_* (and its redundant five permutations) SSRs were absent in the assembled transcriptome. Of the possible 252 unique tetranucleotide‐motif repeats, only 99 were identified in the *C. kousa* transcriptome. The eSSRs found in the transcriptome showed frequencies similar to the eSSRs with primers, in the repeat‐length manner and repeat‐motif manner (Appendix S3: Figure C1).

### 3.2 SSR genotyping and spatial population indices

Our initial data exploration of the egSSR dataset pointed to no clonal MLGs present in the dataset. We also observed violations of the Hardy–Weinberg equilibrium (HWE), likely stemming from the range of sampling time and wide geographic origins of the *C. kousa* accessions (Figure 1; Appendix S3: Figure C2; Appendix S1). In our *C. kousa* collection, only few SSRs were required to capture the MLGs present (Appendix S3: Figure C3). Index of Association score for the egSSR dataset did not agree with the species outcrossing reproduction model, likely due to skewing the dataset with more homozygous and less diverse eSSRs (Appendix S3: Figure C4; Table 2: *N*_L_; *H*_O_). Finally, the low amplitudes (low variability) of the pairwise linkage disequilibrium index (${\bar{r}}_{d}$) implied that the SSRs used in the study were dispersed throughout the *C. kousa* genome, albeit with varying distances between one another (Appendix S3: Figure C5).

### 3.3 Mantel test for isolation by distance, AMOVA, and hierarchical fixation indices

**FIGURE 2:**
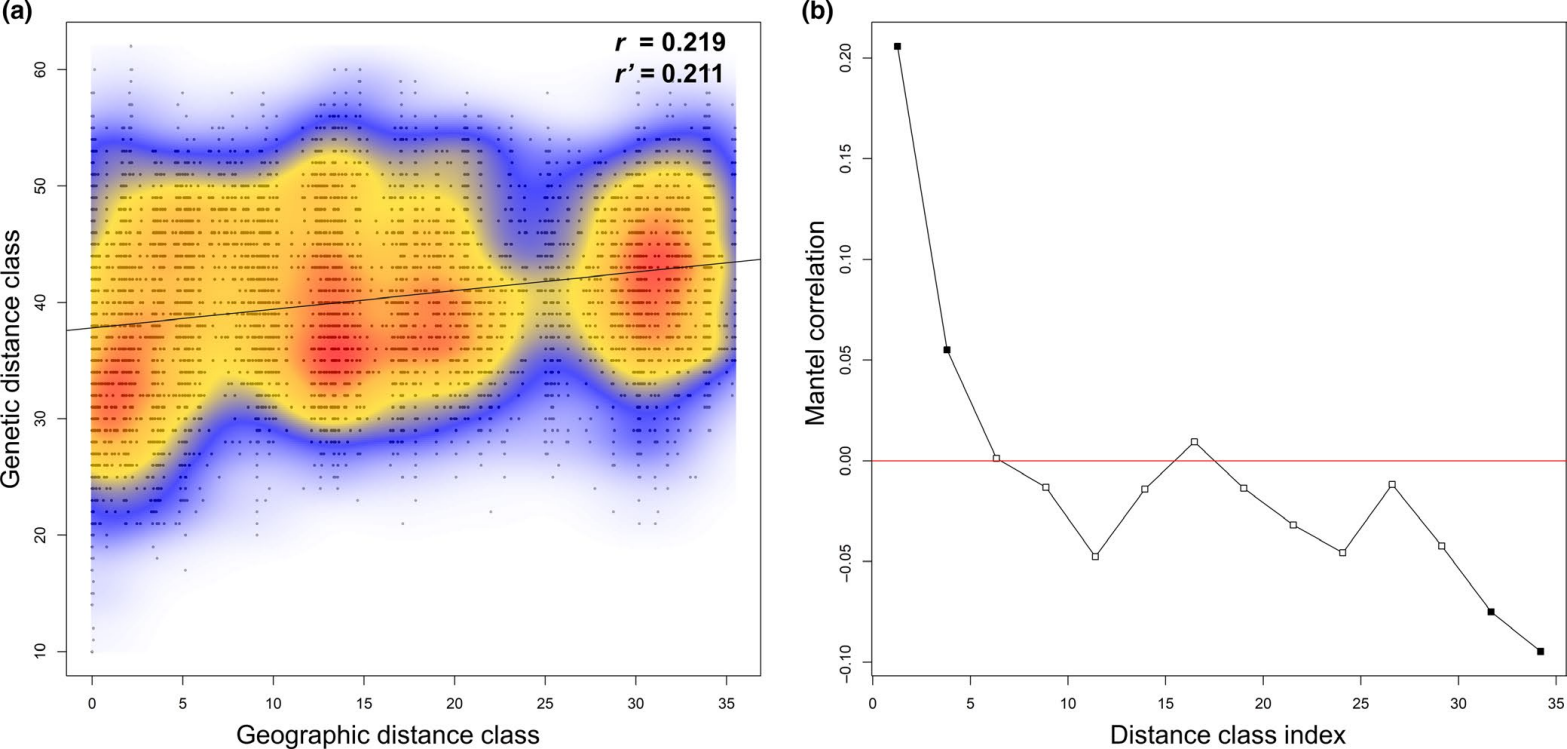
Mantel (a) and Mantel correlogram (b) tests for the isolation‐by‐distance analyses of the *Cornus kousa* egSSR dataset. Data from genotyping the gDNA collection were investigated for the correlation between geographic and genetic distances using packages MASS, ade4, and vegan in R with 1,000 permutations. Correlograms marked with solid symbols are significant at *p* < .001

Results of the Mantel test for isolation by distance indicated that about 5% of the genetic variance was explained by the geographic distance (Mantel's *r* = .219; *p* < .001). Standardizing the geographic distance matrix by the year of sample collection (partial Mantel test) did not significantly change the results (*r* versus *r*′ index values; Figure 2a; Appendix S3: Figure C6). Similarly, the relationship of the genetic and geographic distances across space was nonlinear, although the *C. kousa* specimens were increasingly dissimilar with the growing geographic distance between them (Figure 2b). Low amplitude of the Mantel's *r* scores in the correlograms (from about −0.1 to about 0.2) further underscored that the spatial impact on the population structure of our *C. kousa* collection was not strong (Figure 2b).

Partitioning of the molecular variance was investigated using AMOVA. The proportion of total molecular variance in the egSSR dataset among the populations was about 9.3%; the proportion of the molecular variance partitioned among the individuals and within the populations about 59.3%. As expected for an outcrossing species, an overwhelming majority of the variance was retained within individuals, yet evidence of population structure was detected regardless of the SSRs used (Table ST1).

Analyses of the hierarchical fixation indices (Tables 1 and 2) suggested the existence of population structure for our *C. kousa* collection. All three subpopulations had private alleles, with “China” displaying the highest proportion, with relation to total alleles detected, and the highest allelic richness. Contrastingly, the “Japan” subpopulation had the highest estimate for the number of effective alleles and Shannon's information index. All three subpopulations showed very close values of MLG diversity index, fixation (population inbreeding) index, Simpson's index, high genetic diversity, and the observed heterozygosity indices (Table 1). When analyzed with regard to the SSR loci used (Table 1), we found overall about 9 alleles per locus and about 4 effective alleles per locus. An overall deficit of the observed versus expected heterozygosity implied a population structure within our *C. kousa* collection. Moderate allele fixation, high differentiation, and—correspondingly—departures from HWE toward the excess of homozygotes (inbreeding) were observed (Table 2). This was also reflected in the values of Index of Association and linkage disequilibrium (Table 1) and could be attributed to higher homozygosity of the eSSRs, as expected. Finally, an intensive overall gene flow was estimated for our collection, even after removing the loci with the values flagged as outliers (unusually low or high; 1 locus at either extreme). Comparison of the *F*_ST_ (fixation index; 0.09) and *R*_ST_ (*F*_ST_ analogue based on allele repeat numbers) indicated that mutation was not the major driving force in the *C. kousa* differentiation (Table 2). Only minor differences were observed between indices calculated for eSSR and gSSR, in agreement with the expectations for the genome parts they target, respectively.

### 3.4 Population structure analyses

**FIGURE 3:**
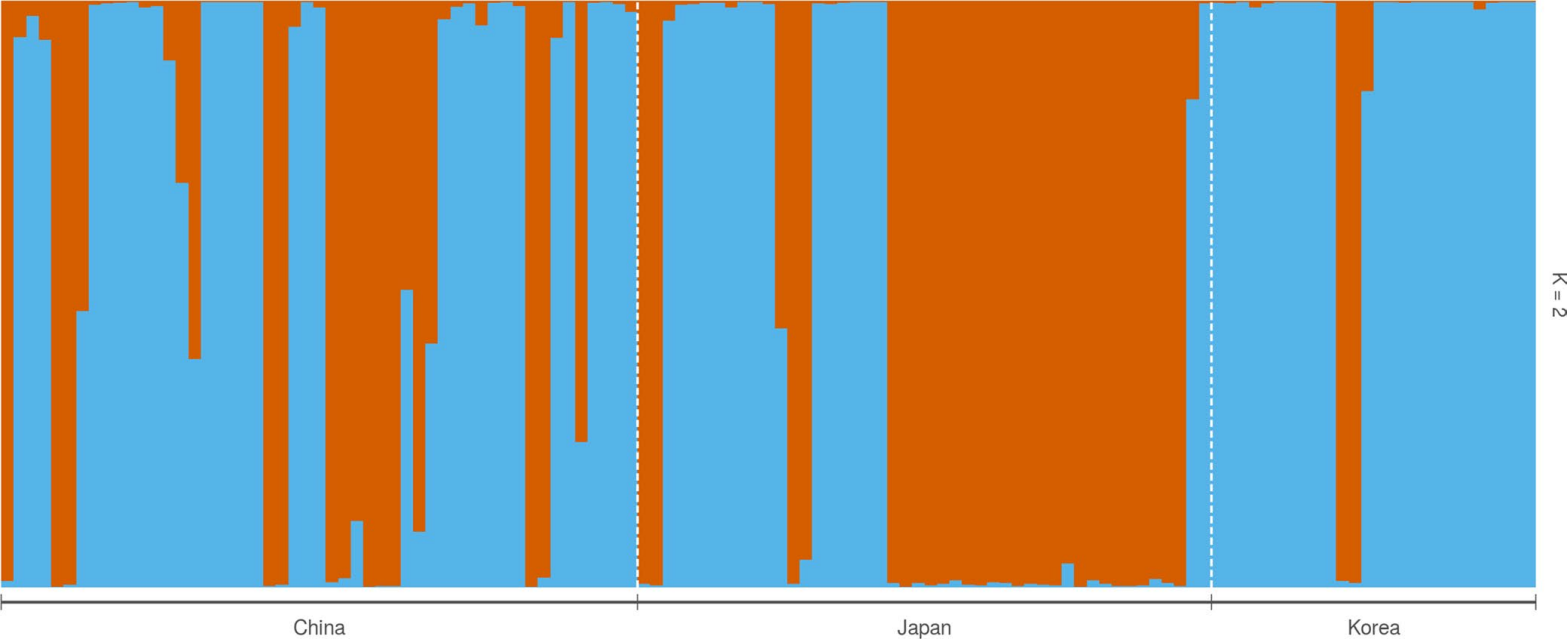
Bayesian clustering of the *Cornus kousa* datasets using structure and Evanno method. Presented are the results for the egSSR genotyped dataset. structure was run for *K* = 1 through 5, using 30 independent Markov chains, with burn‐in of 250,000 and runs of 750,000 steps. Presented are the Bayesian probabilities results for *K* = 2. We visualized the structure results using Evanno's method with PopHelper version 1.0.10 (Evanno et al., 2005; Francis, 2017) to determine the optimal number of clusters. PopHelper was also used to merge the runs of the 30 output files

Several independent analyses indicated the existence of population structure in the collection of *C. kousa*. Results of the Bayesian clustering of the egSSR dataset (structure; Figure 3) indicated two genetically distinct clusters in the genotyped *C. kousa* collection. structure analysis indicated that the predefined subpopulations underwent intensive migration and admixture (Figure 3; Appendix S3: Figure C7). We then analyzed the factors driving the *C. kousa* population structure by iterative independent removal of the predefined populations or the inferred clusters (ObStruct *R* ^2^; Table ST2). The variability observed among 30 independent Markov chains was negligible. Removing “Korea” decreased the overall *R* ^2^ the most, indicating that this subpopulation contributed the most to the signal for population structure. On the other hand, removing “China” caused an increase in *R* ^2^, indicating that this was the most heterogeneous subpopulation harboring individuals with mixed ancestries. Removing the inferred populations in succession resulted in comparable *R* ^2^ values changes, indicating that both the BLUE (mostly present in “China” and “Korea”) and ORANGE inferred clusters (mostly present in the “Japan” subpopulation) contributed comparably to structure within the data (Figure 3; Table ST2).

**FIGURE 4:**
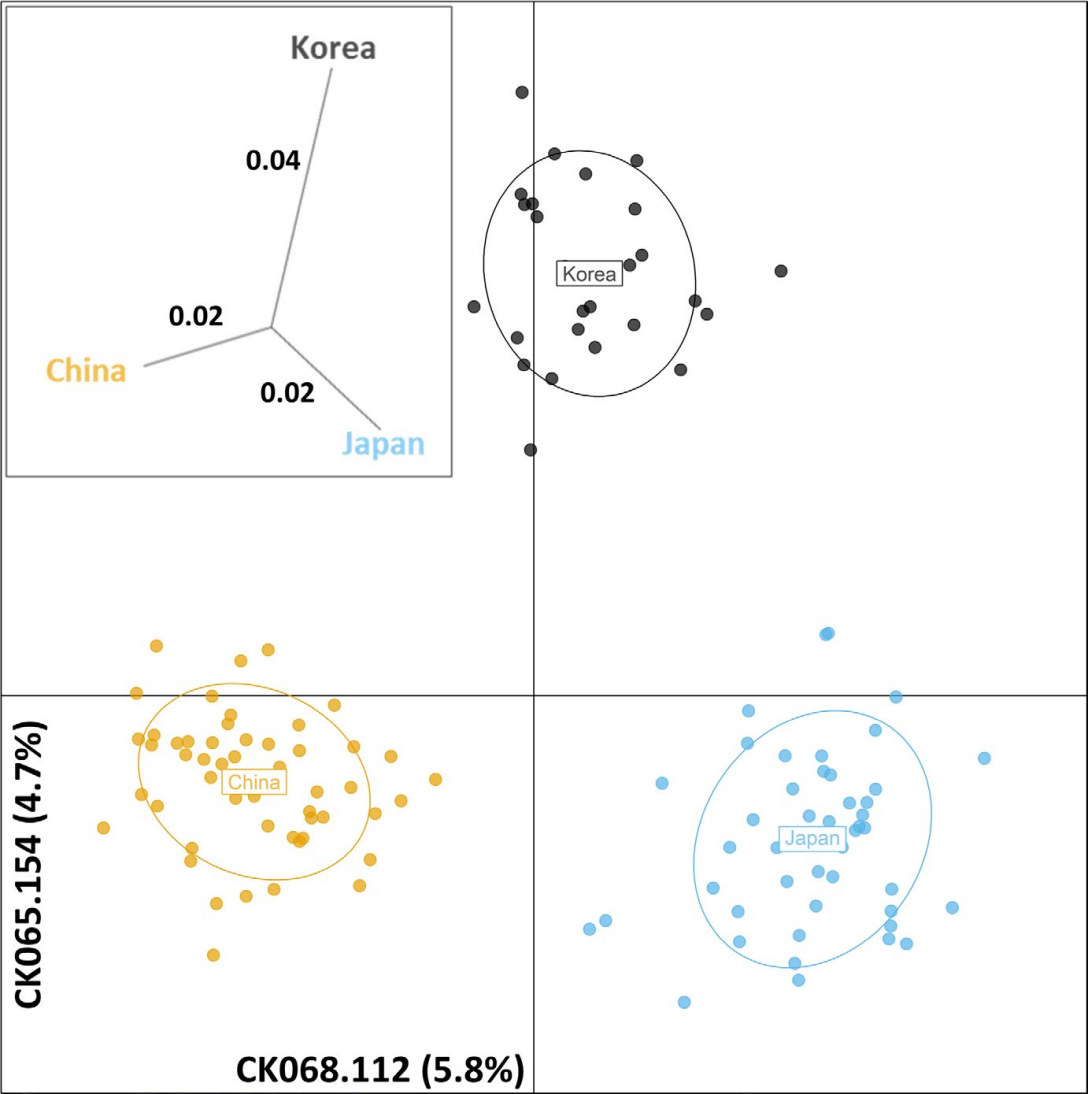
Discriminant analyses of the principal component (DAPC) analysis of the *Cornus kousa* datasets. Data from the combined egSSR dataset were investigated for their molecular variance partitioning using packages ade4 and adegenet in R/RStudio with the number of retained principal components (PCA eigenvalues) optimized and cross‐checked over 1,000 permutations of the dataset. The alleles explaining the most variance are indicated on respective axes, with their % contributions in parenthesis. Insert: Unrooted neighbor‐joining tree of pairwise genetic distances (*F*_ST_, Nei, 1972) among the populations of *C. kousa* analyzed in this study

The multivariate analysis (DAPC) of the egSSR dataset placed the three analyzed subpopulations of *C. kousa* with roughly comparable distances from one another (Figure 4). Country of origin of a given subpopulation influenced its relative placement, in agreement with other analyses in this study. In addition, the DAPC analysis pointed toward a “ghost” unsampled ancestral population for the extant *C. kousa* in its native range (Figure 4; Appendix S3: Figure C8). The subpopulation dendrogram of the genetic distances suggested a grouping congruent with the Bayesian clustering. The genetic distances calculated this way presented an overall low and comparable distances among the subpopulations of *C. kousa* (Inserts: Figure 4; Appendix S3: Figure C8). Collectively, several independent analyses pointed toward existence of *C. kousa* population structure. Genotyping using gSSR and eSSR markers resulted in subtle differences in the placement of subpopulations and/or individuals, and in the genetic distances captured.

### 3.5 Demographic and evolutionary history of *C kousa*

Implementation of the BOTTLENECK program using the applicable mutation models T.P.M. and S.M.M. provided no evidence of a population size variation, and the minor heterozygosity excess/deficiency in few loci lacked significance (data not shown). A normal L‐shaped distribution of each meta‐population was detected with strong statistical support, thus documenting lack of population bottleneck (data not shown).

**FIGURE 5:**
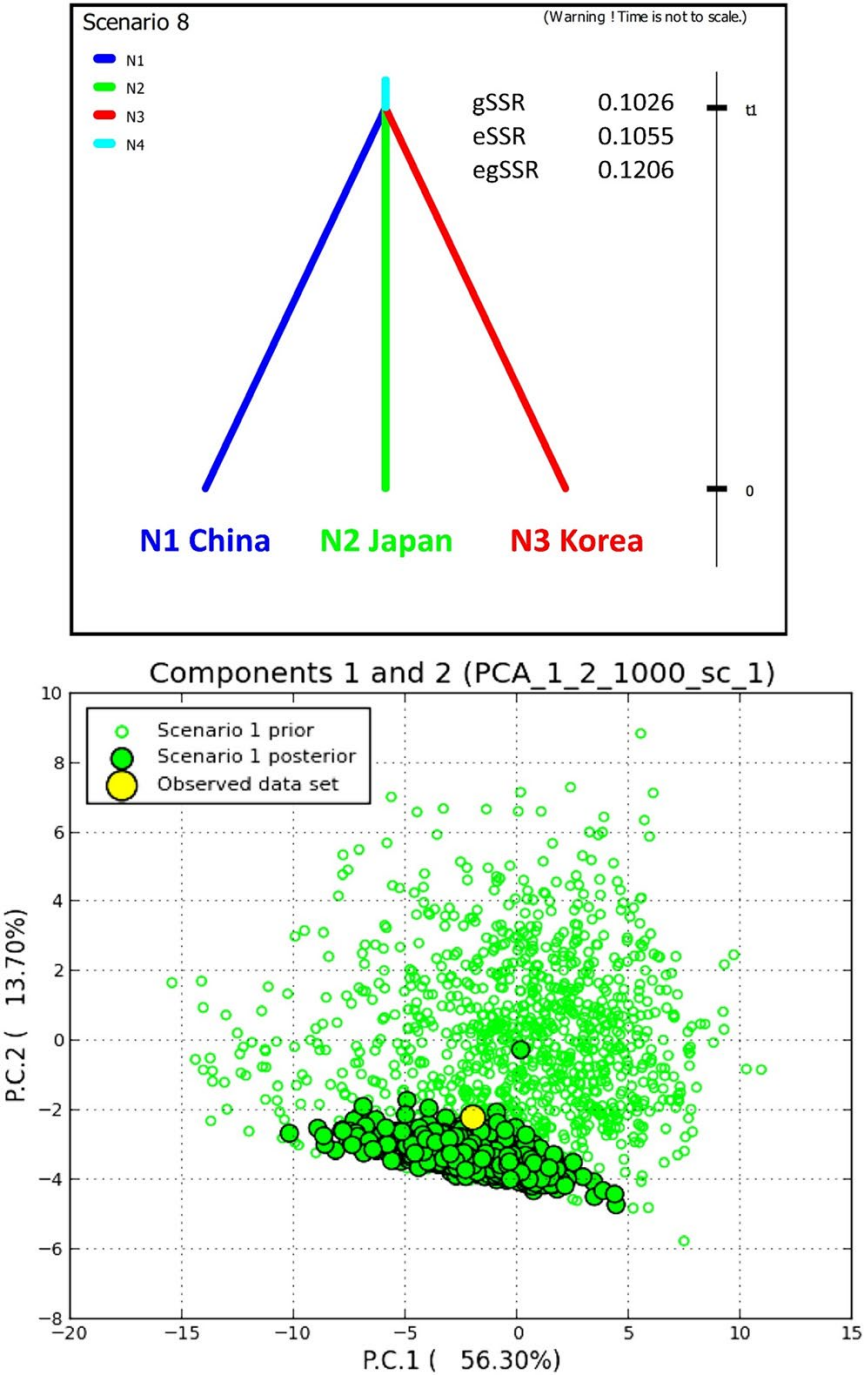
The accepted DIYABC evolutionary scenario for *Cornus kousa*. In total, 35 hypothetical evolutionary scenarios were tested, with the subpopulations of *C. kousa* from China (N1), Japan (N2), and Korea (N3) sampled at *t*0 (present time). Scenario 8 (presented in the top panel) gained good support; it depicts a simultaneous triple split of the originating unsampled “ghost” population (N4) at *t*1 (in the coalescent). Model‐checking analyses of the closest 1% of the simulated prior and posterior datasets under this scenario using PCA supported this scenario choice (bottom panel). Posterior logistic regression relative probabilities for this scenario from the comparison of 35 scenarios for each dataset are tabulated in the insert, top panel (gSSR, eSSR, egSSR, respectively)

Evolutionary scenario reconstruction lent the highest support to a concurrent triple split of the originating unsampled “ghost” population (Appendix S2). When the lack of support for population bottleneck was regarded, the dataset supported the “ghost” population split into “China” and “Japan” subpopulations that later admixed to give rise to “Korea” subpopulation (Appendix S2). The scenario with the “ghost” population that was split three concurrent ways consistently ranked among the top‐three highest‐supported scenarios in all the datasets analyzed this way (eSSR, gSSR, and egSSR). Considering all the DIYABC results, we accepted this as the most likely evolutionary scenario for *C. kousa* (Figure 5). The related posterior parameter estimates for the egSSR dataset under this scenario suggested a recent split that occurred around 220 generations ago (CI 95% 40–613) and estimated the species mutation rate at about 3 × 10^–4^ per locus per generation (CI 95% 1.5 × 10^–4^ to 7.1 × 10^–4^). The effective population sizes were estimated as several thousands of individuals (CI 95% from several hundred to around 10,000 individuals), whereas the originating “ghost population” size was estimated at about 5,000 individuals (CI 95% from 900 to 10,000) (Table ST3).

Further analyses of the phylogenetic patterns in our *C. kousa* genotyped egSSR dataset were carried out using the program SPAGeDi, by permuting the allele sizes among alleles within locus. The mean *R*_ST_ value over all loci (0.077 ± 0.02; CI 95% 0.042 to 0.121) did not differ from the observed *R*_ST_ value of 0.10. It was also not larger than the observed *F*_ST_ value of 0.09 (*P*_obs>exp_ = 0.1027; Table 2). This implied lack of the phylogeographic signal within populations,* that is*, the alleles were not more related within populations than among populations. Comparatively, permuting the slopes of subpopulation geographic distances versus the genetic distances, in both linear and logarithmic forms, showed significant differences in the means of the permuted values versus observed (slope linear: observed 0.0001; mean permuted: −2.6E−05 ± 7.4E−05; CI 95% −0.00022 to 9.07E−05; *P*_obs>exp_ = 0.016; slope ln transformed: observed 0.17; mean permuted −0.035 ± 0.124; CI 95% −0.357 to 0.176; *P*_obs>exp_ = 0.028). This observation implied a phylogeographic signal among populations, that is, alleles were more related between nearby populations of *C. kousa* than between distant populations. Finally, this analysis also estimated the value of (*δμ*)^2^ parameter as 1.831. After implementing the DIYABC mutation rate of ~0.0003 per locus per generation, it resulted with the *C. kousa* populations split time estimated at about 305 generations into the coalescent.

## 4 DISCUSSION

In this study, we aimed to investigate the genetic diversity and the population structure of *C. kousa,* a popular ornamental tree species of economic importance, in its native range. In order to reach that goal, we applied genotyping using genomic (Wadl et al., 2009, 2010; Wadl, Wang, Scheffler, et al., 2008; Wadl, Wang, Trigiano, et al., 2008) and newly developed genic microsatellites (gSSRs and eSSRs, respectively), capillary electrophoresis, and a host of population genetic analyses of gSSR and eSSR datasets, and the combined egSSR dataset. Our sample collection points to the high scientific value of the generally underappreciated and underexplored resources of herbaria (preserved) and arboreta (living sample depositories).

Microsatellites or SSRs have been widely used in the population genetics and evolution studies of plants (Ellis & Burke, 2007; Raquin et al., 2008), and other organisms (Bhargava & Fuentes, 2010; Kim et al., 2019; Raquin et al., 2008; Rubinsztein et al., 1995). Both gSSRs and eSSRs are in common use. Despite their inherent differences in the targeted parts of the genome, they tend to provide congruent and complementary insights into the species diversity and histories (Nowicki, Zhao, et al., 2019 and examples discussed therein; Ellis & Burke, 2007; Bhargava & Fuentes, 2010). The emerging consensus of the SSR studies is that the eSSRs capture comparatively somewhat lower genetic distances among the populations than the gSSRs. The eSSRs allow for greater cross‐amplification due to the more conserved character of the transcriptome, thus allowing to assess species differentiation and delimitation with more reliability than gSSRs (Ellis & Burke, 2007; Nowicki, Zhao, et al., 2019). In particular, the advent of next‐generation sequencing accelerated the SSR development, with eSSR‐based research gaining great momentum. Here, the assembly of the next‐generation sequencing data is quicker and easier by not requiring prior genome knowledge and by the comparatively more conserved character of the transcriptome (Ellis & Burke, 2007; Gibbons et al., 2009). Ease of the eSSR generation is illustrated in this study, with just one available assembled transcriptome (Yu et al., 2017) generating eSSRs in abundance. From the initially tested 50 eSSRs, 47 amplified the test gDNA sample “Morris43.” Of those, 16 eSSRs presented inconsistent results and owing to this were discarded from further analyses. From the remaining 31 eSSRs, 17 were selected based on our stringent criteria and used to genotype the collection of *C. kousa*. In our study, the *C. kousa* subpopulations presented a largely similar overall picture of the species diversity irrespective of the SSR type used. We did observe the evidence for population structure and modest level of diversity in the outcrossing *C. kousa* on the basis of geographic origin of a given subpopulation. In particular, high values of the *C. kousa* genetic diversity index (μH*_E_*) are encouraging information for the species diversity preservation (Yuan, Fang, Liu, & Fu, 2019). We inferred the existence of diverged gene pools in *C. kousa*. This is a valuable information for the commercial cultivars’ development with regard to possible deployment or pyramiding of the desired utility traits (Hagan et al., 1998; Merkle, Andrade, Nairn, Powell, & Maynard, 2007; Molnar et al., 2017). In our study, the SSRs were distributed across the entire genome. This can guide the future QTL studies or construction of the genetic maps (Wadl et al., 2011; Wang et al., 2009).

Of the Cornaceae, *C. kousa* diversity and population genetics have rarely been investigated. Interspecific hybridization with the North America‐native *C. florida* (upon orchestration of their flowering) has been successful. Yet, clear species‐level differences were observed in the chloroplast DNA (Nowicki et al., 2018) and using genotyping by sequencing (Pfarr et al., 2018). Kousa dogwood possesses a number of desirable utility traits, such as enhanced pathogen resistance (Hagan et al., 1998; Li et al., 2007; Merkle et al., 2007; Thurn et al., 2018) and abiotic stress tolerance (Cappiello & Shadow, 2005). Furthermore, a longer and later flowering time than the native North American *C. florida* (Cappiello & Shadow, 2005) increases the ornamental, and thus, economical, value of the *kousa*‐derived commercial cultivars. Indeed, the estimated number of available commercial cultivars of *C. kousa* equals, if not exceeds, those of *C. florida* (Cappiello & Shadow, 2005; Wadl, Windham, et al., 2014). In addition, hybrid cultivars with *C. capitata* Wall. ex Roxb., *C. nuttallii*, and *C. florida* (dubbed *C. *×* rutgersensis*) have been released, following the market demand, and broadening the available selections (Cappiello & Shadow, 2005; Mattera, 2016; Mattera et al., 2015). Experimental hybrids with other *Cornus* species were also procured, although no commercial cultivars were released (*C. elliptica* (Pojark.) QY Wadlford and *C. hongkongensis* (Hemsl.) Hutch.; Shearer & Ranney, 2013a, Shearer & Ranney, 2013b). Several studies investigated anthocyanins produced by *C. kousa* for potential pharmaceuticals (Seeram, Schutzki, Chandra, & Nair, 2002; Vareed, Reddy, Schutzki, & Nair, 2006). Species diversity was mostly addressed at the phenotypical level following the Korean conservation efforts (Song, Goo, Han, Yang, & Park, 2006). Korean forest managers used the same species as a model for effective family size optimization while maintaining both the genetic diversity and seedling production (Kang & Lee, 2008). Previous studies used intersimple sequence repeat markers (ISSRs) to investigate 13 wild local Korean accessions to distinguish differently colored bract specimens on mainland Korea from the Oenaro Island plants (Kim, Kwon, Park, Oh, & Choi, 2006) and 12 local Chinese populations of the var. *chinensis* (Yuan et al., 2019). In this regard, especially considering the high economic potential for the species, our study presents the very first species‐wide investigation of the genetic diversity and population structure in the noncultivated *C. kousa*. Despite not having a rigorous sampling scheme, the collection used for this investigation covered the species native range, reflecting its genetic diversity. The results reported herein can be regarded as an initial assessment of the *C. kousa* genetic richness until more robust collections are studied. The general conclusion of the diversity related to the plant country of origin has important implications for the existing and future breeding programs involving *C. kousa* when trait pyramiding is intended. The analyzed specimens of *C. kousa* cover the range of approximately 10 generations but are unlikely to incur any major bias. Moreover, the estimates of the SSR mutation rate fell within the expected range. The SSRs are favored for their relatively high mutation rate of 10^–6^ to 10^–3^ mutations per locus per generation (Bhargava & Fuentes, 2010; Raquin et al., 2008). Taken together, any bias based on the age of the samples would have a miniscule probability to manifest within our *C. kousa* collection, let alone skew the results of the analyses. This fact was also reflected in our partial Mantel tests where the standardization of isolation by distance with the age of sample essentially did not change the outcomes.

The evolutionary history of *C. kousa* awaits investigations. Recent fossil‐based (Atkinson, 2018) and transcriptome‐based inferences (Yu et al., 2017) of Cornales species were only partially congruent. In both studies, the big‐bracted dogwoods diverged about 45 MYA, but the following divergence inferences differ. The fossil record (Atkinson, 2018) points to the divergence of both *C. kousa* and *C. canadensis* from a common ancestor with *C. florida* undergoing the subsequent speciation at about 24 MYA. On the other hand, the transcriptome study (Yu et al., 2017) placed the *C. canadensis* as the base for the subsequent big‐bracted dogwood monophyletic splits. The only two confirmed polyploid dogwoods are *C. canadensis* and *C. unalaschkensis* Ledeb. (Bai, Alverson, Follansbee, & Waller, 2012; Dermen, 1932; Love, 1982; Shearer & Ranney, 2013a, 2013b). Hastened molecular evolution of the genome of the polyploid *C. canadensis* was postulated (Yu et al., 2017). Hence, that species may be the common ancestor of the extant assembly of big‐bracted dogwood species. Our study allowed a glimpse into the *C. kousa* phylogeographic evolutionary history: Analyses pointed to the “China” subpopulation as possibly the most ancestral one. Contrastingly, the DIYABC analyses indicated that the three sampled regions originated from a “ghost” (unsampled) population possibly following the typical invasion and expansion mode. The DIYABC analyses congruently with the SPAGeDi (δμ)^2^ estimated the divergence time following the last glacial maximum geological models for the species native region (Scotese, 2014). It could follow the previous presence of land masses that later submerged, transforming into the present time Yellow Sea and Sea of Japan region (Appendix S3: Figure C9; Scotese, 2014), had indeed been populated by the *C. kousa* common ancestral population.

Comparison of the permuted *R*_ST_ and *F*_ST_ values showed no significant differences. This evidenced lack of phylogeographic patterns within our *C. kousa* collections, as only the genetic structure due to allelic identity remained (Hardy & Vekemans, 2015). Such null hypothesis (*R*_ST_ = *F*_ST_) implied that the *C. kousa* mutation rate is negligible compared with the species migration rate. By contrast, permuting the linear or ln slope of population geographic distances showed the phylogeographic signal extant among the *C. kousa* populations. This implied that differentiation between distant populations is greater than between nearby ones. Such conclusion was also supported by small the yet significant score of the isolation‐by‐distance test. As such, the differentiation of *C. kousa* may be attributed to the recent divergence driven by the species geographic expansion. The divergence was not mitigated by the intensive gene flow which sustained the modest within‐population genetic diversity.

## 5 CONCLUSIONS

Available herbarium and arboretum resources enabled the assessment of the species diversity and evolutionary history of the Asian dogwood, *C. kousa*, a tree species of economic importance in the nursery industry. Genotyping analyses of our collection with the newly developed eSSRs and the available gSSRs yielded insights into the species diversity. Analyses of the species evolutionary history using the accrued genotyping datasets pointed to an unsampled ancestor population, possibly now extinct, as per the phylogeography. Recognition of the species‐distinct genetic pools related to the plant country of origin can be helpful in the ongoing and future breeding projects for the commercial cultivars, and for the possible germplasm conservation efforts. On the practical side, this information may allow for breeding/pyramiding utility traits, including hybridization with the North America‐native dogwoods.

## CONFLICT OF INTEREST

No conflict of interest has been declared by the author(s).

## AUTHOR CONTRIBUTION

**Marcin Nowicki:** Conceptualization (equal); Data curation (equal); Formal analysis (equal); Investigation (equal); Methodology (equal); Project administration (equal); Validation (equal); Visualization (equal); Writing‐original draft (equal); Writing‐review & editing (equal). **Logan C. Houston:** Data curation (equal); Formal analysis (equal); Investigation (equal); Writing‐review & editing (equal). **Sarah L. Boggess:** Data curation (equal); Formal analysis (equal); Project administration (equal); Resources (equal); Visualization (equal); Writing‐review & editing (equal). **Anthony S. Aiello:** Resources (equal); Writing‐review & editing (equal). **Miriam Payá‐Milans:** Software (equal); Validation (equal); Writing‐review & editing (equal). **Margaret E. Staton:** Resources (equal); Software (equal); Validation (equal). **Mitsuhiro Hayashida:** Resources (equal); Writing‐review & editing (equal). **Masahiro Yamanaka:** Resources (equal); Writing‐review & editing (equal). **Shigetoshi Eda:** Resources (equal); Writing‐review & editing (equal). **Robert N. Trigiano:** Funding acquisition (lead); Investigation (equal); Project administration (equal); Resources (equal); Supervision (equal); Writing‐original draft (equal); Writing‐review & editing (equal).

## Supporting information

Appendix S1

Appendix S2

Appendix S3

Table S1‐S3

## Data Availability

The raw data that support the findings of this study are published in Appendix S1. The *C. kousa* transcriptome is published at http://doi.org/10.1371/journal.pone.0171361.
