# Supplementary material for: Species diversity and phylogeography of Cornus kousa (Asian dogwood) captured by genomic and genic microsatellites
Source: Ecol Evol. 2020 Jul 11;10(15):8299–312. doi: 10.1002/ece3.6537 (PMC7417245; doi:10.1002/ece3.6537)
Supplement: Supplementary file 2 — Appendix S2 [file ECE3-10-8299-s003.pdf]

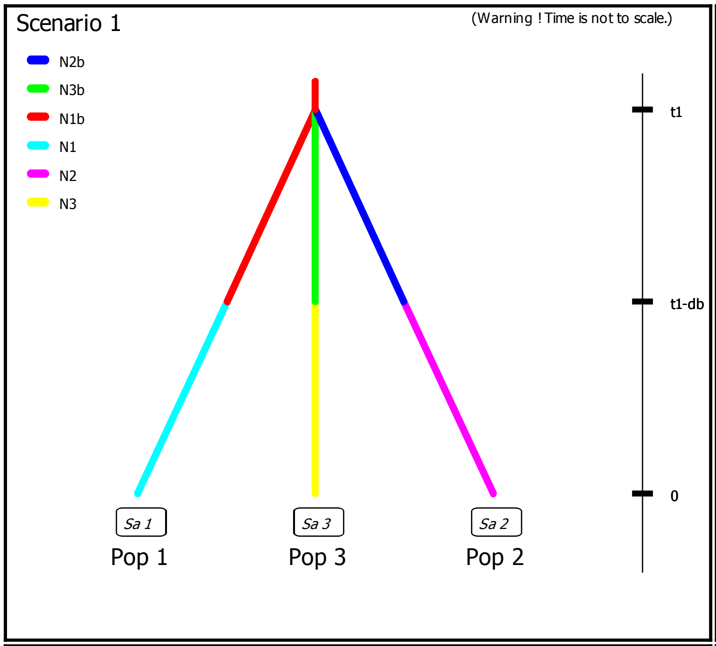

Scenario 1
(Warning ! Time is not to scale.)
N2b
N3b
N1b
N1
N2
N3
t1
t1-db
0
Sa 1
Sa 3
Sa 2
Pop 1
Pop 3
Pop 2

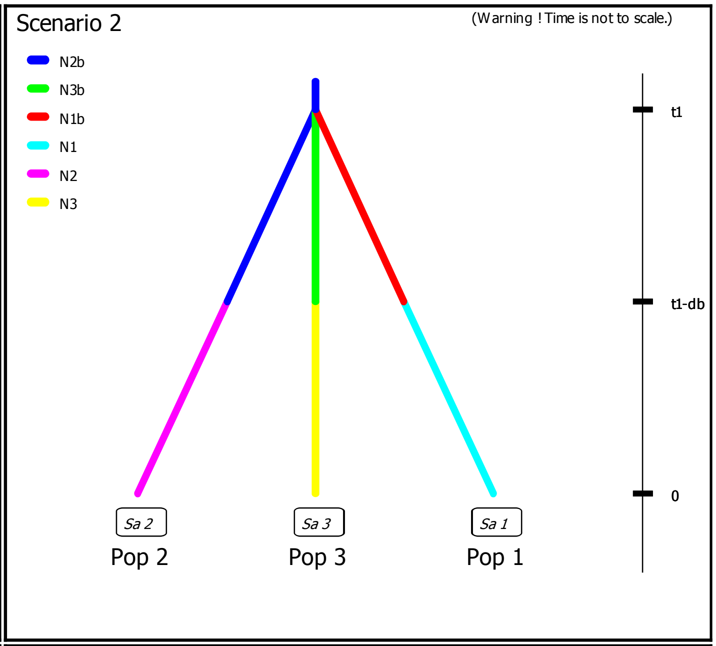

Scenario 2
(Warning ! Time is not to scale.)
N2b
N3b
N1b
N1
N2
N3
t1
t1-db
0
Sa 2
Sa 3
Sa 1
Pop 2
Pop 3
Pop 1

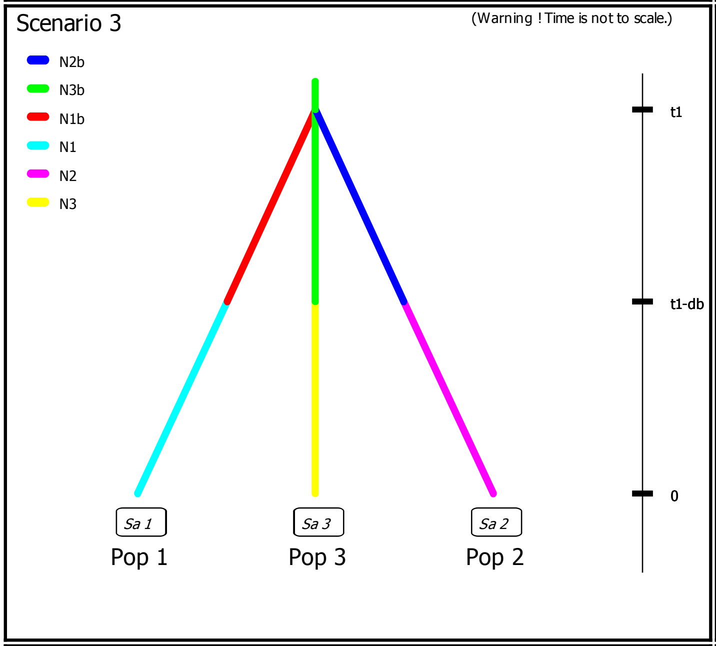

Scenario 3
(Warning ! Time is not to scale.)
N2b
N3b
N1b
N1
N2
N3
t1
t1-db
0
Sa 1
Sa 3
Sa 2
Pop 1
Pop 3
Pop 2

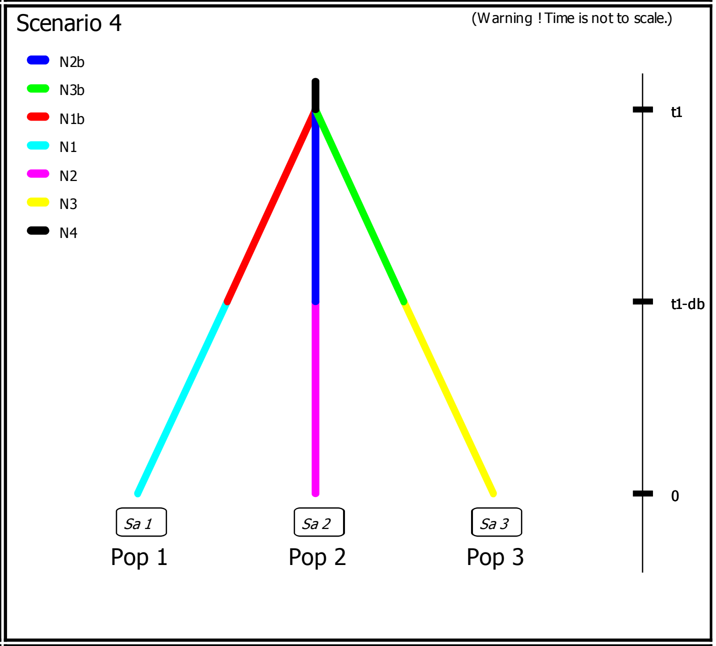

Scenario 4
(Warning ! Time is not to scale.)
N2b
N3b
N1b
N1
N2
N3
N4
t1
t1-db
0
Sa 1
Sa 2
Sa 3
Pop 1
Pop 2
Pop 3

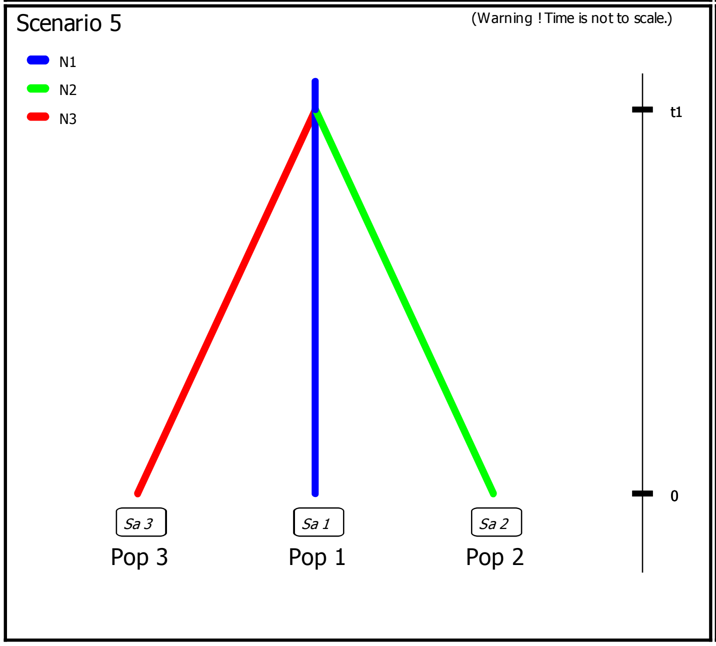

Scenario 5
(Warning ! Time is not to scale.)
N1
N2
N3
t1
0
Sa 3
Sa 1
Sa 2
Pop 3
Pop 1
Pop 2

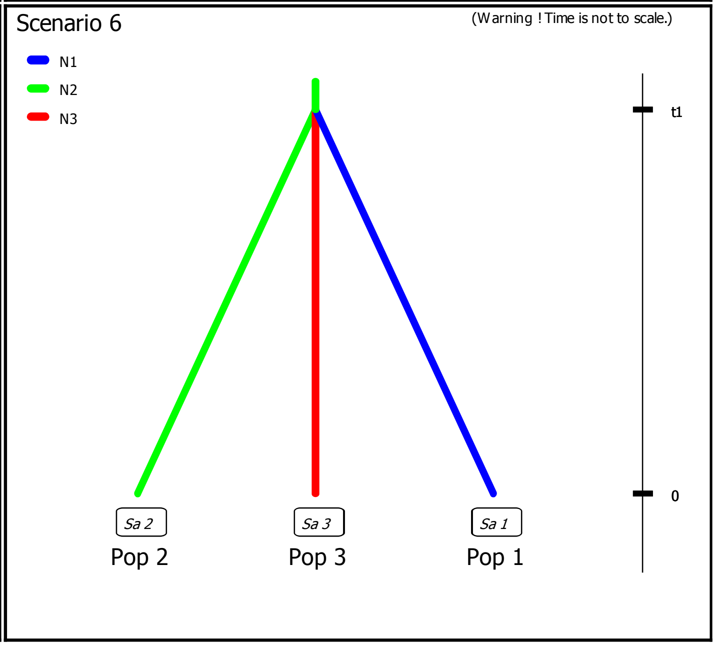

Scenario 6
(Warning ! Time is not to scale.)
N1
N2
N3
t1
0
Sa 2
Sa 3
Sa 1
Pop 2
Pop 3
Pop 1

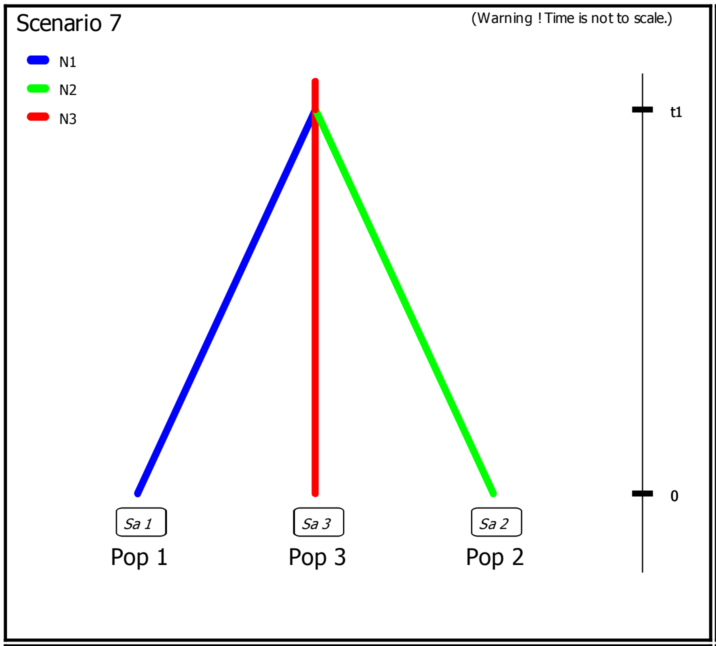
Scenario 7
(Warning ! Time is not to scale.)
N1
N2
N3
t1
0
Sa 1
Pop 1
Sa 3
Pop 3
Sa 2
Pop 2

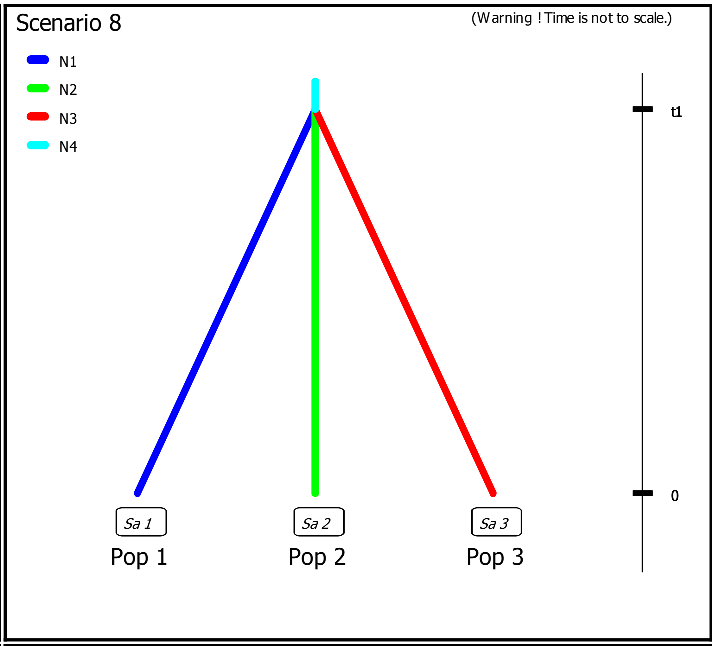
Scenario 8
(Warning ! Time is not to scale.)
N1
N2
N3
N4
t1
0
Sa 1
Pop 1
Sa 2
Pop 2
Sa 3
Pop 3

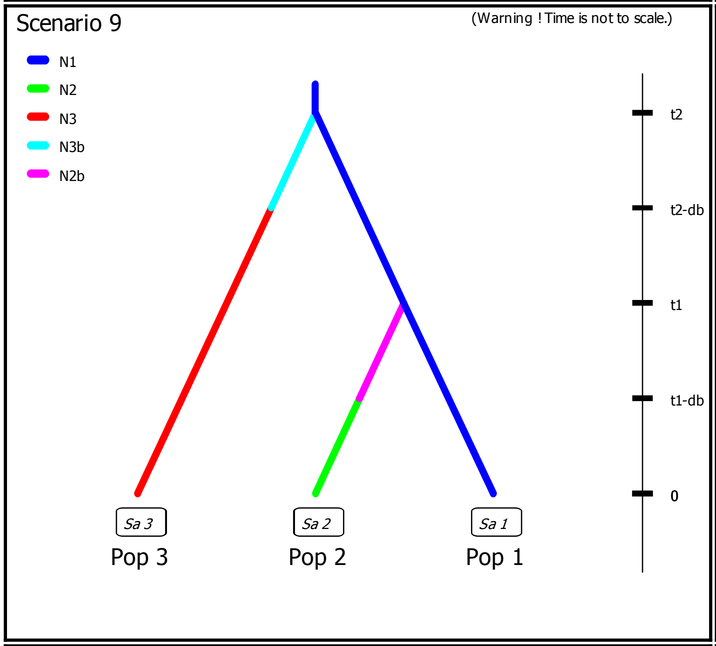
Scenario 9
(Warning ! Time is not to scale.)
N1
N2
N3
N3b
N2b
t2
t2-db
t1
t1-db
0
Sa 3
Pop 3
Sa 2
Pop 2
Sa 1
Pop 1

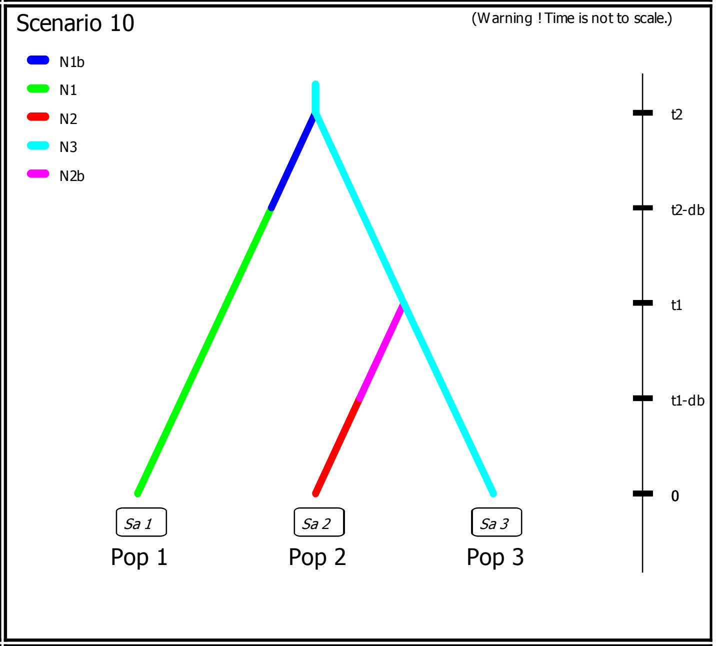
Scenario 10
(Warning ! Time is not to scale.)
N1b
N1
N2
N3
N2b
t2
t2-db
t1
t1-db
0
Sa 1
Pop 1
Sa 2
Pop 2
Sa 3
Pop 3

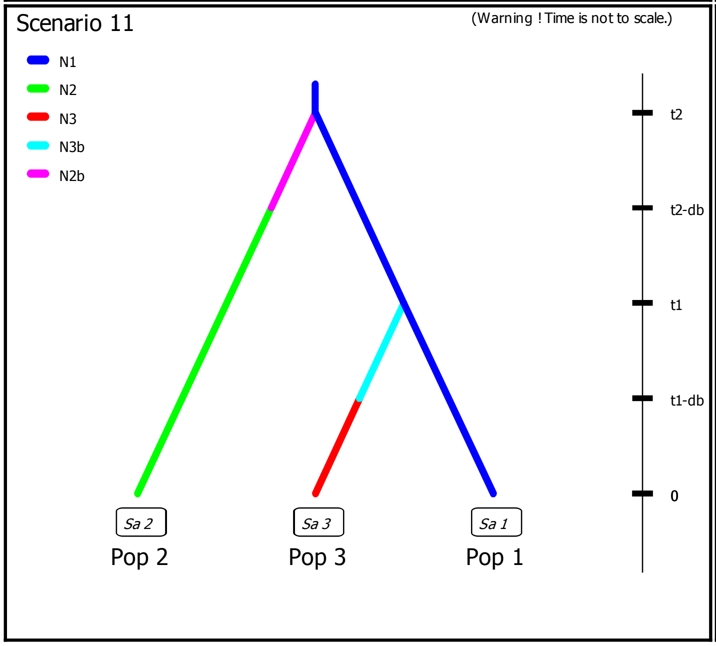
Scenario 11
(Warning ! Time is not to scale.)
N1
N2
N3
N3b
N2b
t2
t2-db
t1
t1-db
0
Sa 2
Pop 2
Sa 3
Pop 3
Sa 1
Pop 1

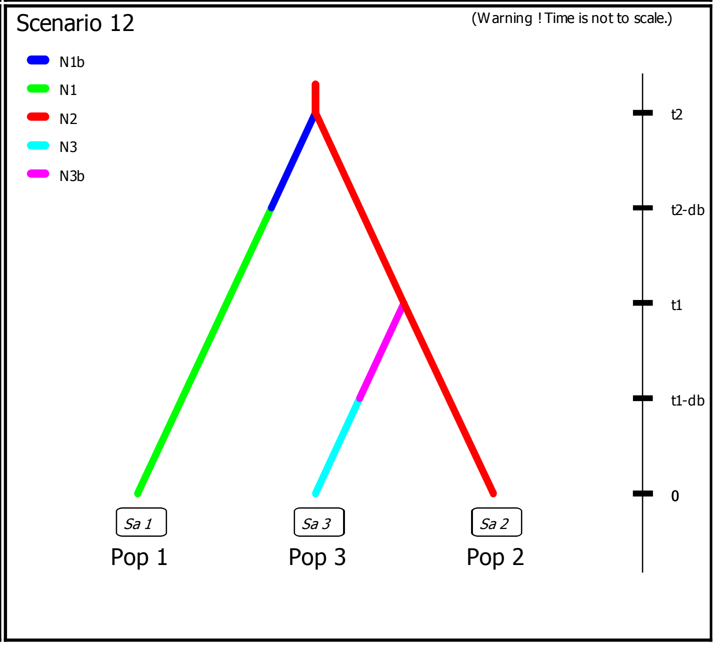
Scenario 12
(Warning ! Time is not to scale.)
N1b
N1
N2
N3
N3b
t2
t2-db
t1
t1-db
0
Sa 1
Pop 1
Sa 3
Pop 3
Sa 2
Pop 2

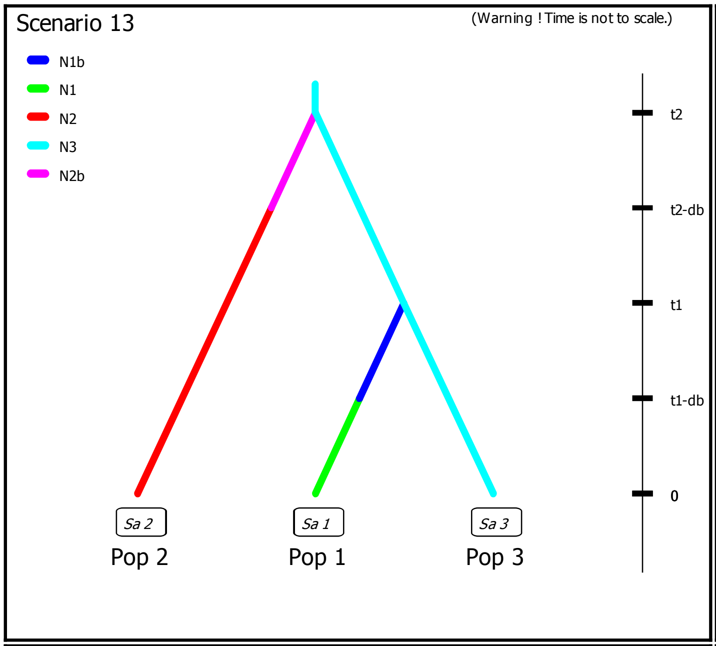
Scenario 13
(Warning ! Time is not to scale.)
N1b
N1
N2
N3
N2b
t2
t2-db
t1
t1-db
0
Sa 2
Pop 2
Sa 1
Pop 1
Sa 3
Pop 3

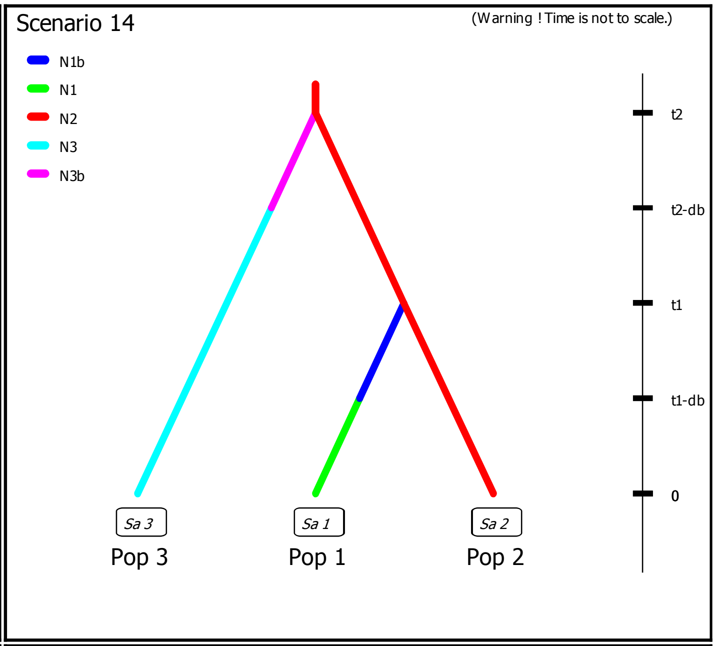
Scenario 14
(Warning ! Time is not to scale.)
N1b
N1
N2
N3
N3b
t2
t2-db
t1
t1-db
0
Sa 3
Pop 3
Sa 1
Pop 1
Sa 2
Pop 2

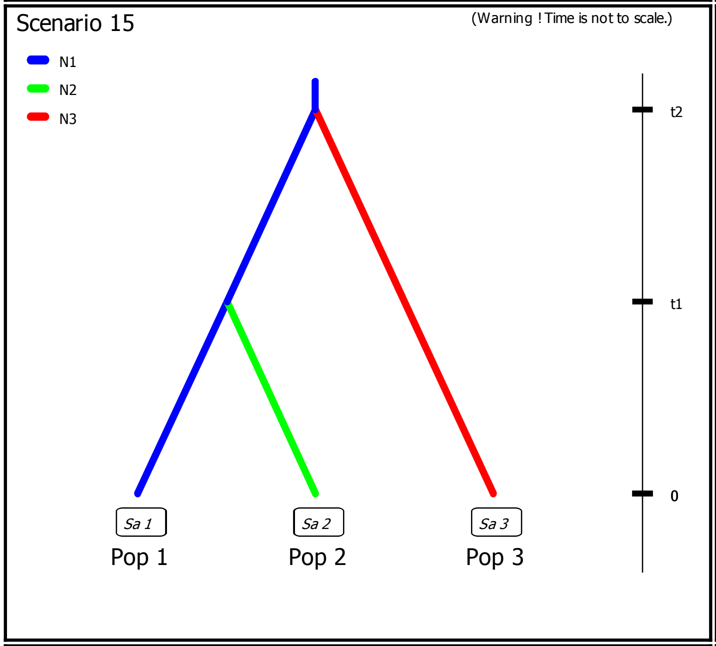
Scenario 15
(Warning ! Time is not to scale.)
N1
N2
N3
t2
t1
0
Sa 1
Pop 1
Sa 2
Pop 2
Sa 3
Pop 3

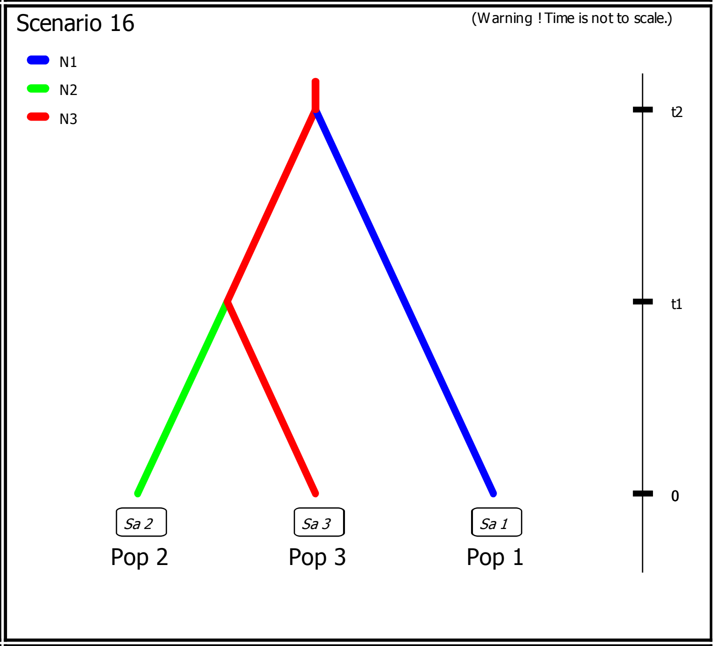
Scenario 16
(Warning ! Time is not to scale.)
N1
N2
N3
t2
t1
0
Sa 2
Pop 2
Sa 3
Pop 3
Sa 1
Pop 1

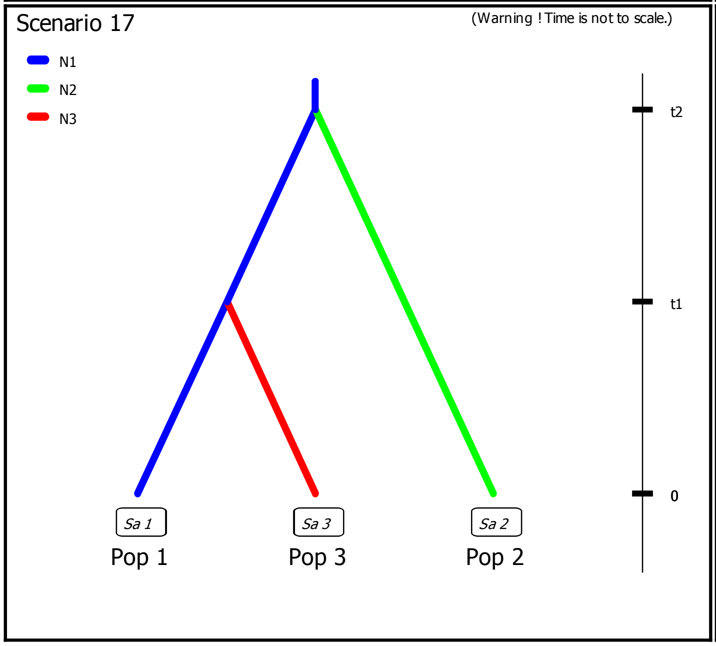
Scenario 17
(Warning ! Time is not to scale.)
N1
N2
N3
t2
t1
0
Sa 1
Pop 1
Sa 3
Pop 3
Sa 2
Pop 2

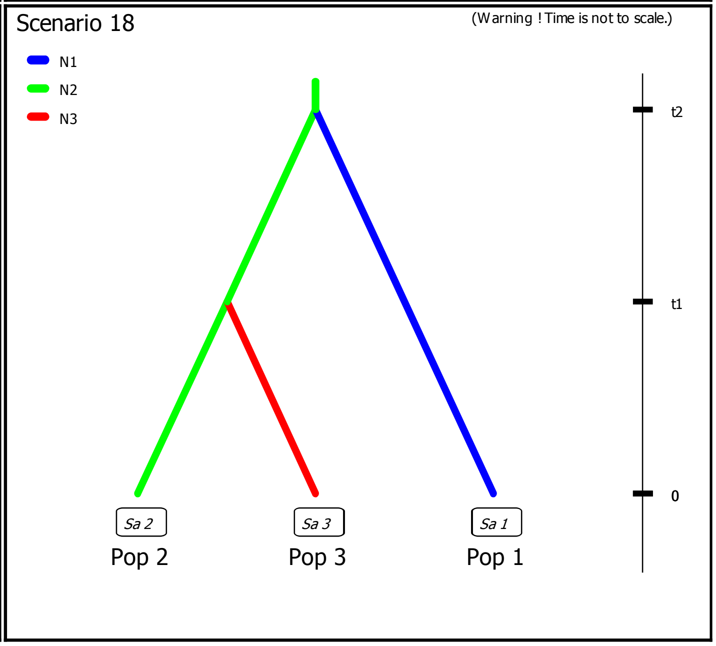
Scenario 18
(Warning ! Time is not to scale.)
N1
N2
N3
t2
t1
0
Sa 2
Pop 2
Sa 3
Pop 3
Sa 1
Pop 1

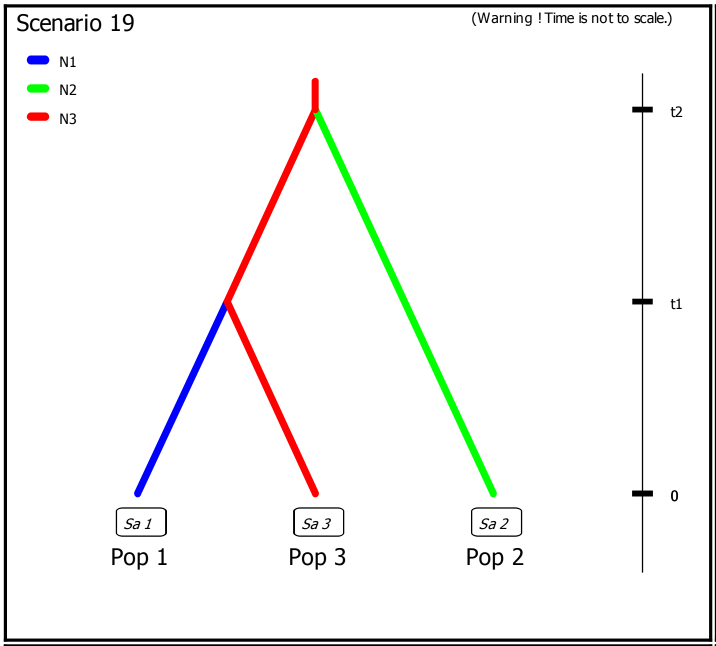
Scenario 19
(Warning ! Time is not to scale.)
N1
N2
N3
t2
t1
0
Sa 1
Sa 3
Sa 2
Pop 1
Pop 3
Pop 2

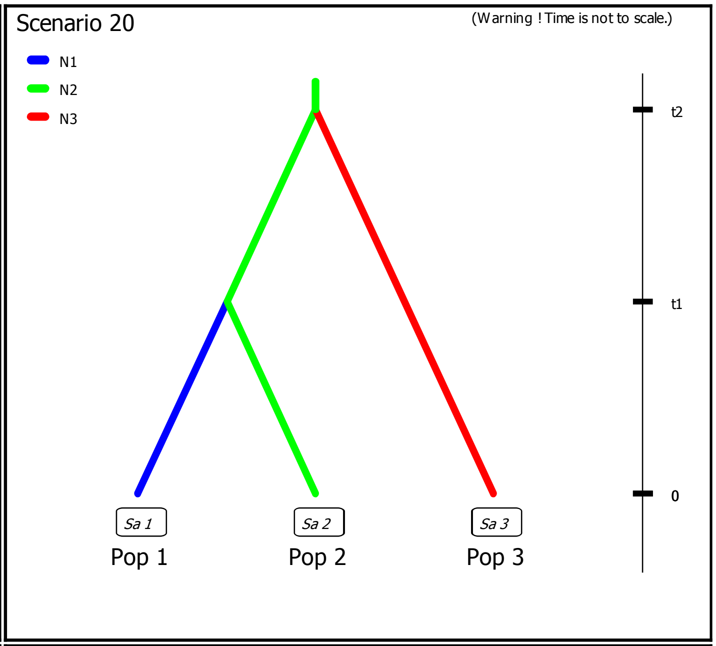
Scenario 20
(Warning ! Time is not to scale.)
N1
N2
N3
t2
t1
0
Sa 1
Sa 2
Sa 3
Pop 1
Pop 2
Pop 3

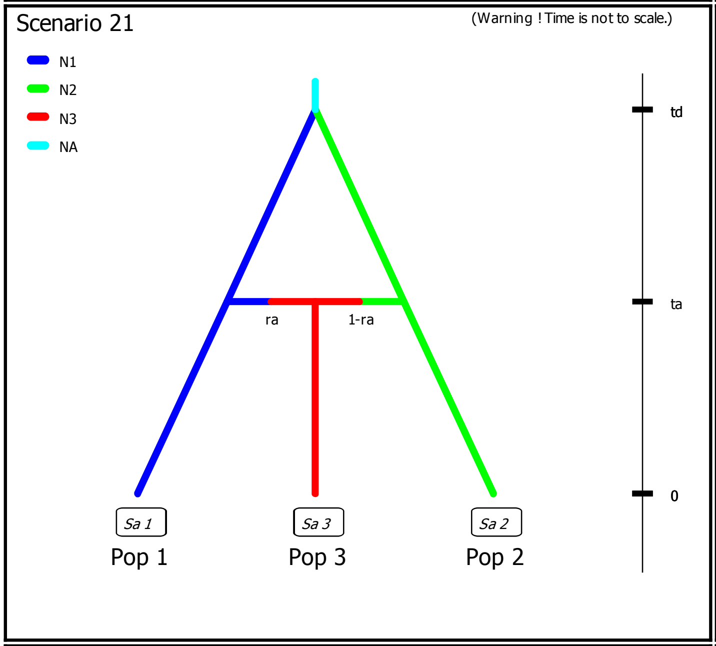
Scenario 21
(Warning ! Time is not to scale.)
N1
N2
N3
NA
td
ta
0
ra
1-ra
Sa 1
Sa 3
Sa 2
Pop 1
Pop 3
Pop 2

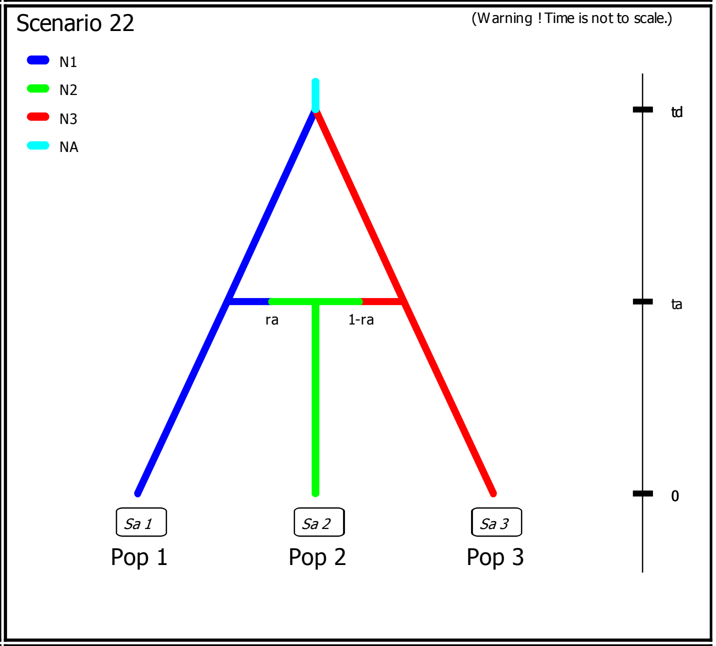
Scenario 22
(Warning ! Time is not to scale.)
N1
N2
N3
NA
td
ta
0
ra
1-ra
Sa 1
Sa 2
Sa 3
Pop 1
Pop 2
Pop 3

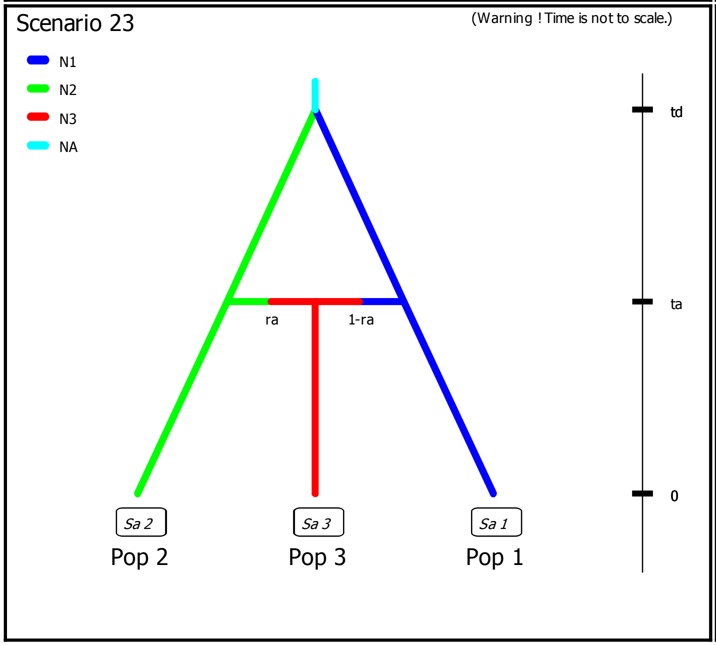
Scenario 23
(Warning ! Time is not to scale.)
N1
N2
N3
NA
td
ta
0
ra
1-ra
Sa 2
Sa 3
Sa 1
Pop 2
Pop 3
Pop 1

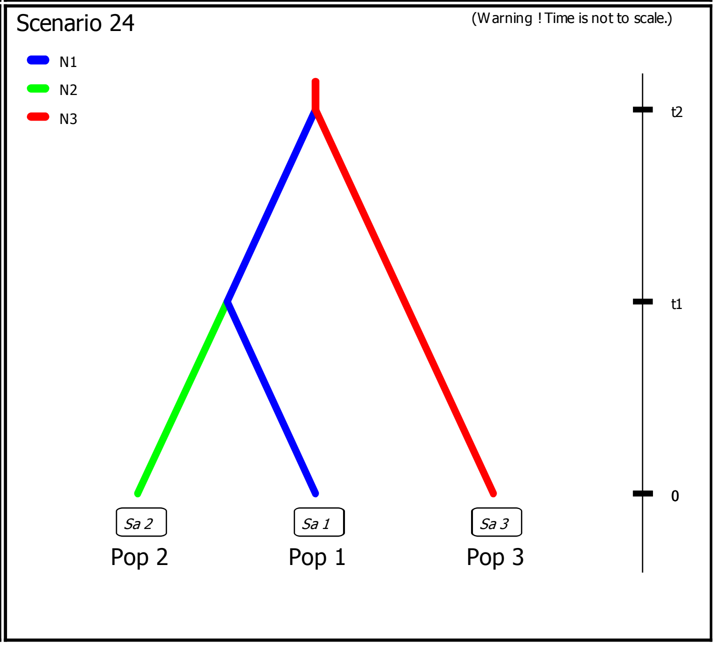
Scenario 24
(Warning ! Time is not to scale.)
N1
N2
N3
t2
t1
0
Sa 2
Sa 1
Sa 3
Pop 2
Pop 1
Pop 3

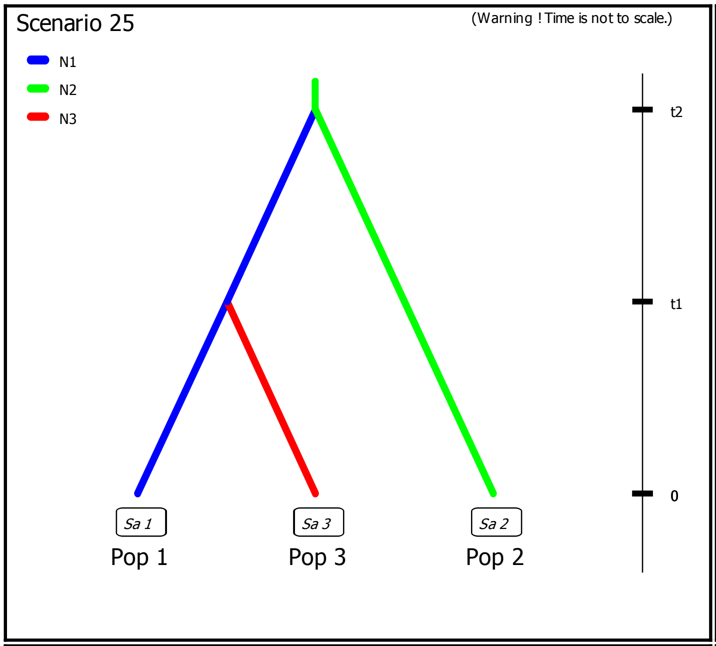
Scenario 25
(Warning ! Time is not to scale.)
N1
N2
N3
t2
t1
0
Sa 1
Pop 1
Sa 3
Pop 3
Sa 2
Pop 2

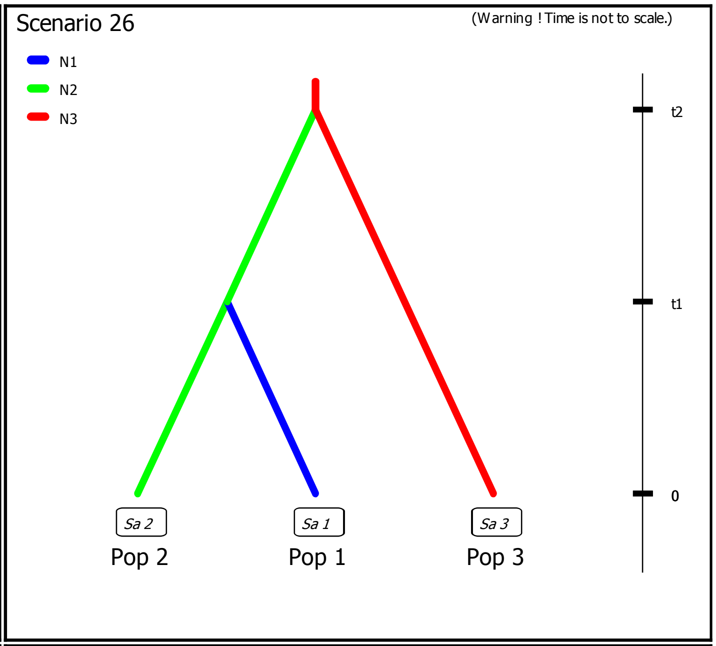
Scenario 26
(Warning ! Time is not to scale.)
N1
N2
N3
t2
t1
0
Sa 2
Pop 2
Sa 1
Pop 1
Sa 3
Pop 3

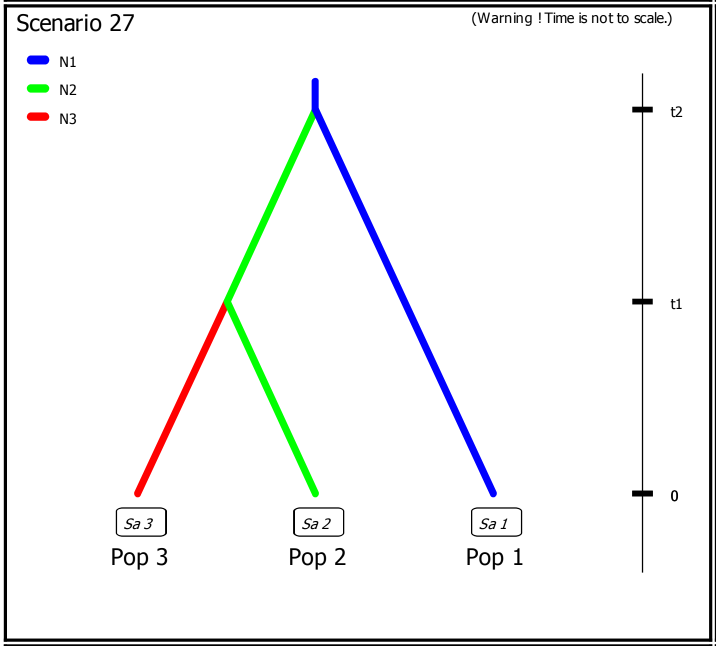
Scenario 27
(Warning ! Time is not to scale.)
N1
N2
N3
t2
t1
0
Sa 3
Pop 3
Sa 2
Pop 2
Sa 1
Pop 1

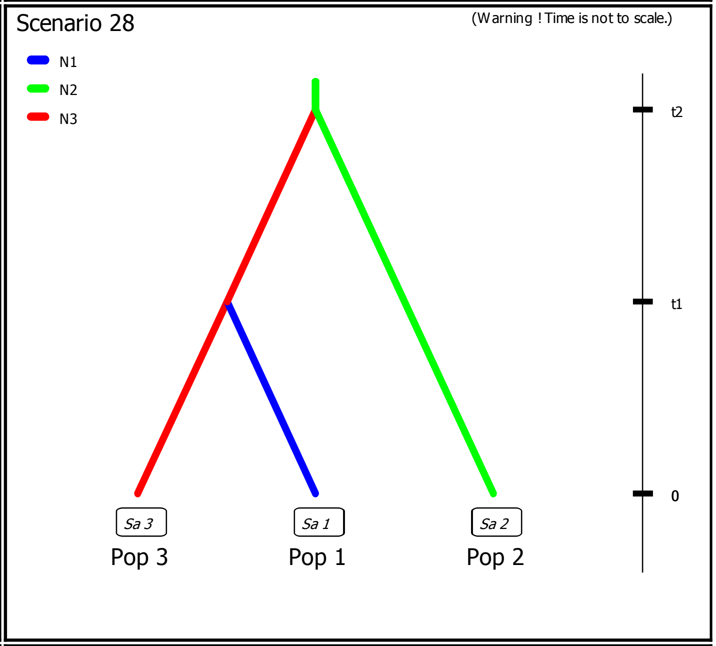
Scenario 28
(Warning ! Time is not to scale.)
N1
N2
N3
t2
t1
0
Sa 3
Pop 3
Sa 1
Pop 1
Sa 2
Pop 2

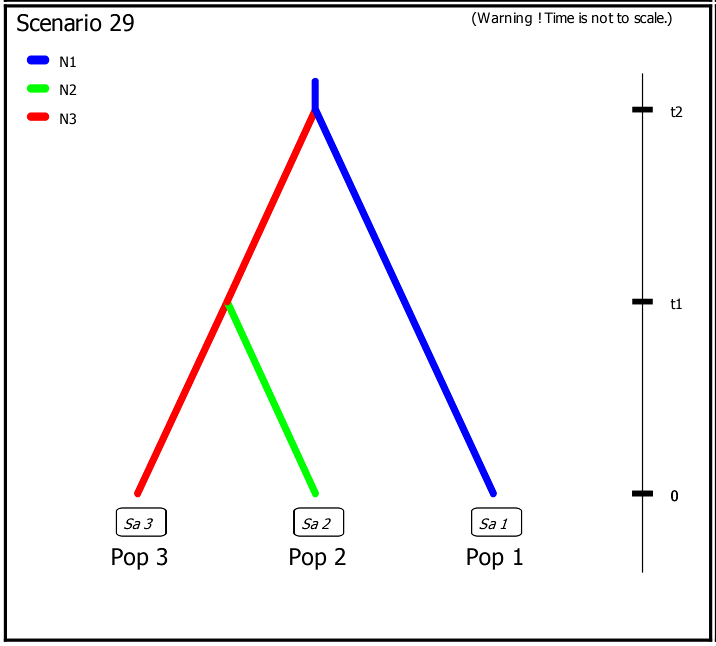
Scenario 29
(Warning ! Time is not to scale.)
N1
N2
N3
t2
t1
0
Sa 3
Pop 3
Sa 2
Pop 2
Sa 1
Pop 1

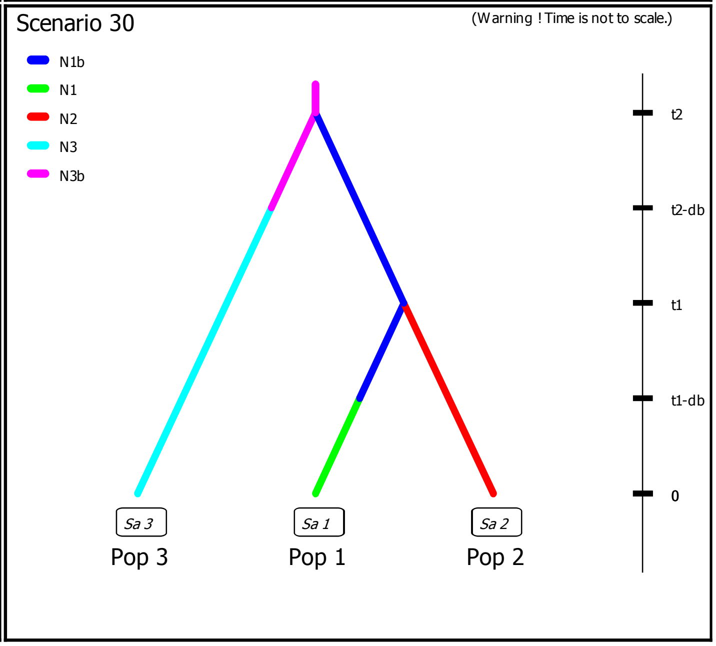
Scenario 30
(Warning ! Time is not to scale.)
N1b
N1
N2
N3
N3b
t2
t2-db
t1
t1-db
0
Sa 3
Pop 3
Sa 1
Pop 1
Sa 2
Pop 2

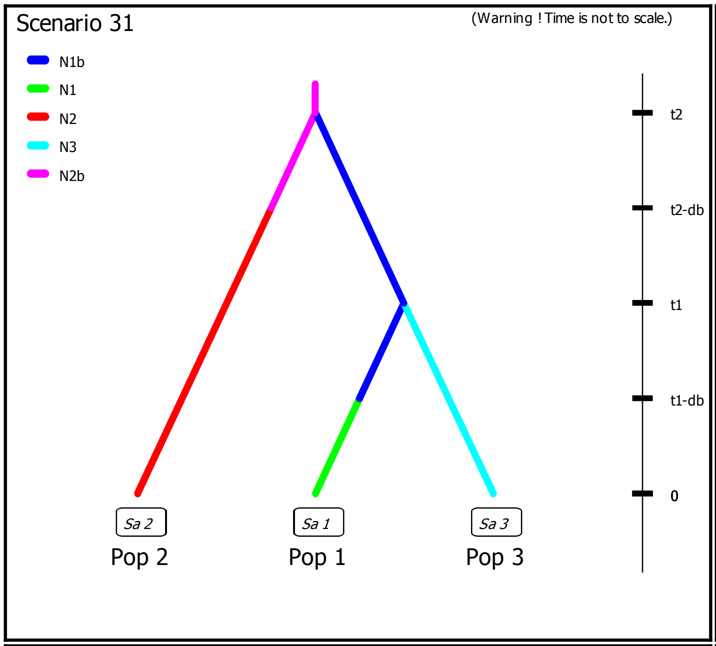
Scenario 31
(Warning ! Time is not to scale.)
N1b
N1
N2
N3
N2b
t2
t2-db
t1
t1-db
0
Sa 2
Pop 2
Sa 1
Pop 1
Sa 3
Pop 3

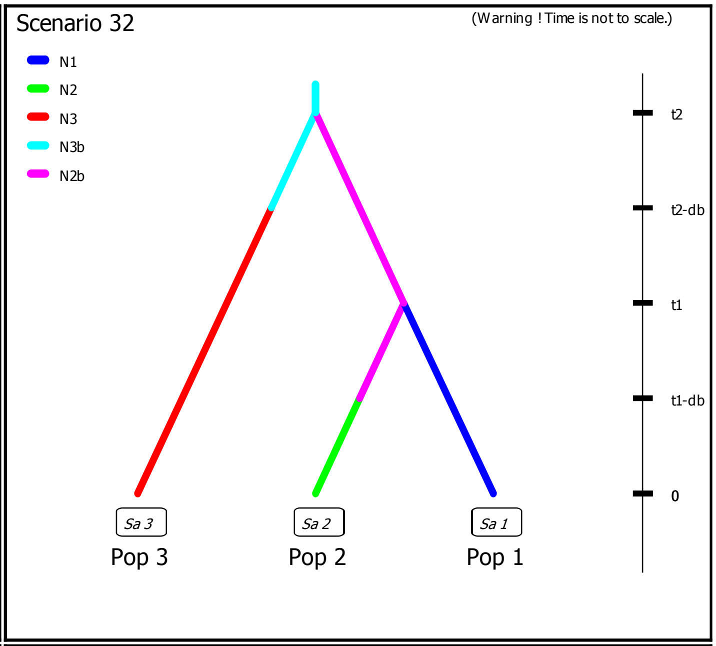
Scenario 32
(Warning ! Time is not to scale.)
N1
N2
N3
N3b
N2b
t2
t2-db
t1
t1-db
0
Sa 3
Pop 3
Sa 2
Pop 2
Sa 1
Pop 1

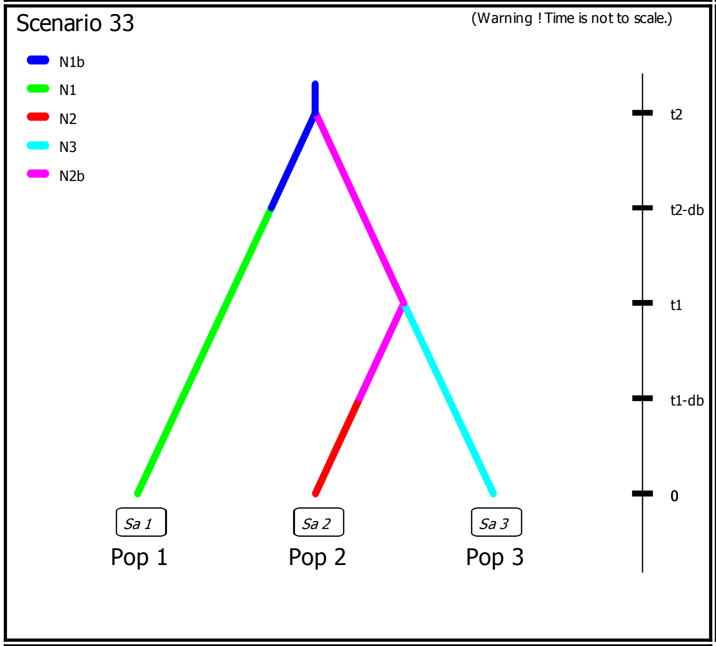
Scenario 33
(Warning ! Time is not to scale.)
N1b
N1
N2
N3
N2b
t2
t2-db
t1
t1-db
0
Sa 1
Pop 1
Sa 2
Pop 2
Sa 3
Pop 3

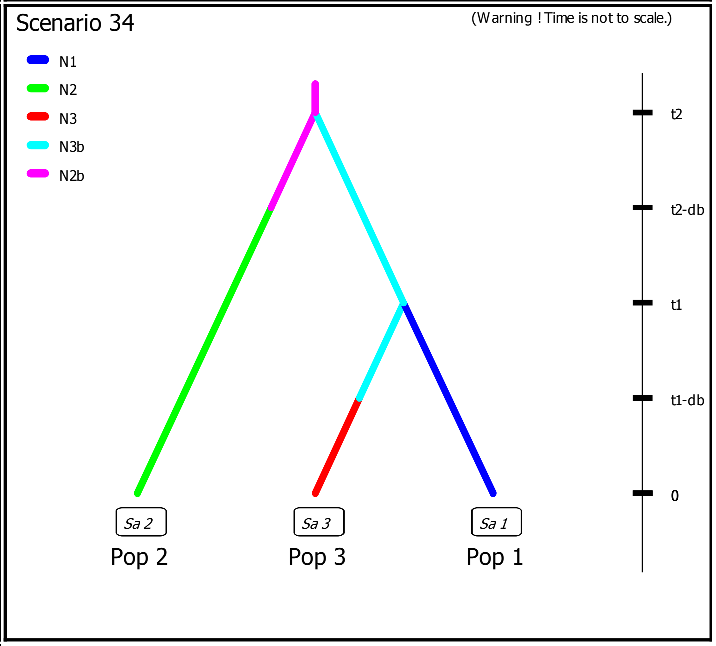
Scenario 34
(Warning ! Time is not to scale.)
N1
N2
N3
N3b
N2b
t2
t2-db
t1
t1-db
0
Sa 2
Pop 2
Sa 3
Pop 3
Sa 1
Pop 1

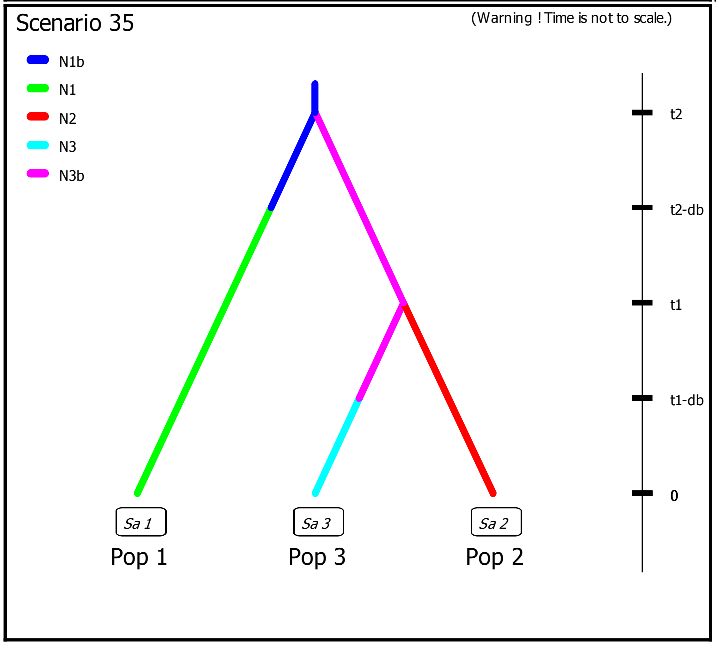
Scenario 35
(Warning ! Time is not to scale.)
N1b
N1
N2
N3
N3b
t2
t2-db
t1
t1-db
0
Sa 1
Pop 1
Sa 3
Pop 3
Sa 2
Pop 2
