# Supplementary material for: Species diversity and phylogeography of Cornus kousa (Asian dogwood) captured by genomic and genic microsatellites
Source: Ecol Evol. 2020 Jul 11;10(15):8299–312. doi: 10.1002/ece3.6537 (PMC7417245; doi:10.1002/ece3.6537)
Supplement: Supplementary file 3 — Appendix S3 [file ECE3-10-8299-s004.docx]

**Appendix C to:** Species diversity and phyleogeography of *Cornus kousa* (Asian dogwood) captured by genomic and genic microsatellites

Figure C1. Genic short sequence repeat markers (eSSRs) discovered in the transcriptome of *Cornus kousa* (Yu et al. 2017).

Figure C2. Hardy-Weinberg equilibrium (HWE) for the *Cornus kousa* datasets.

Figure C3. Genotype accumulation curves (GACs) for the *Cornus kousa* datasets.

Figure C4. Assessment of clonality by Index of Association for the *Cornus kousa* datasets.

Figure C5. Assessment of Linkage Disequilibrium by standardized overall Index of Association $(\bar{r}$_d_) for the *Cornus kousa* datasets.

Figure C6. Mantel (top) and Mantel correlogram (bottom) tests for the isolation-by-distance analyses of the *Cornus kousa* datasets.

Figure C7. Bayesian clustering of the *Cornus kousa* datasets using Structure and Evanno method.

Figure C8. Discriminant Analyses of the Principal Components (DAPC) of the *Cornus kousa* datasets,

Figure C9. Map of the origin of locations of the *Cornus kousa* individuals sampled for the study, during Last Glacial Maximum.


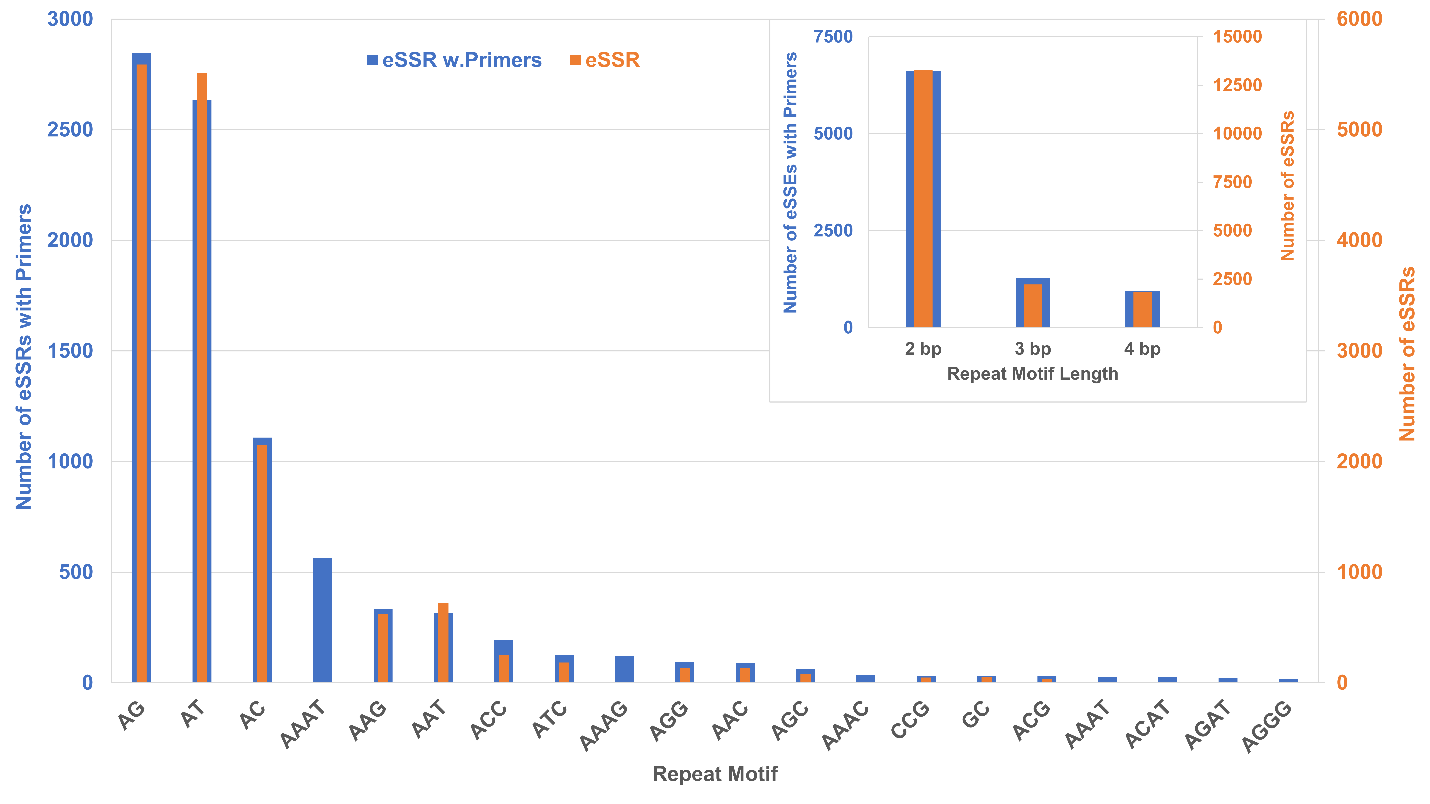


Figure C1. Genic short sequence repeat markers (eSSRs) discovered in the transcriptome of *Cornus kousa* (Yu et al. 2017). Assembled RNAseq reads were screened for presence of di-, tri-, and tetra-motif repeat SSRs. Frequency (counts) of the 20 most-frequent eSSR non-redundant motifs found in the assembled transcriptome are depicted in orange; eSSRs with primers constructed as per our algorithm are shown in blue. Insert: Frequency (counts) of di-, tri-, and tetra-motif repeat eSSRs in the assembled *Cornus kousa* transcriptome. Note: Frequencies of non-redundant tetra-repeat eSSRs were not inspected in the transcriptome assembly before devising the primers for them.


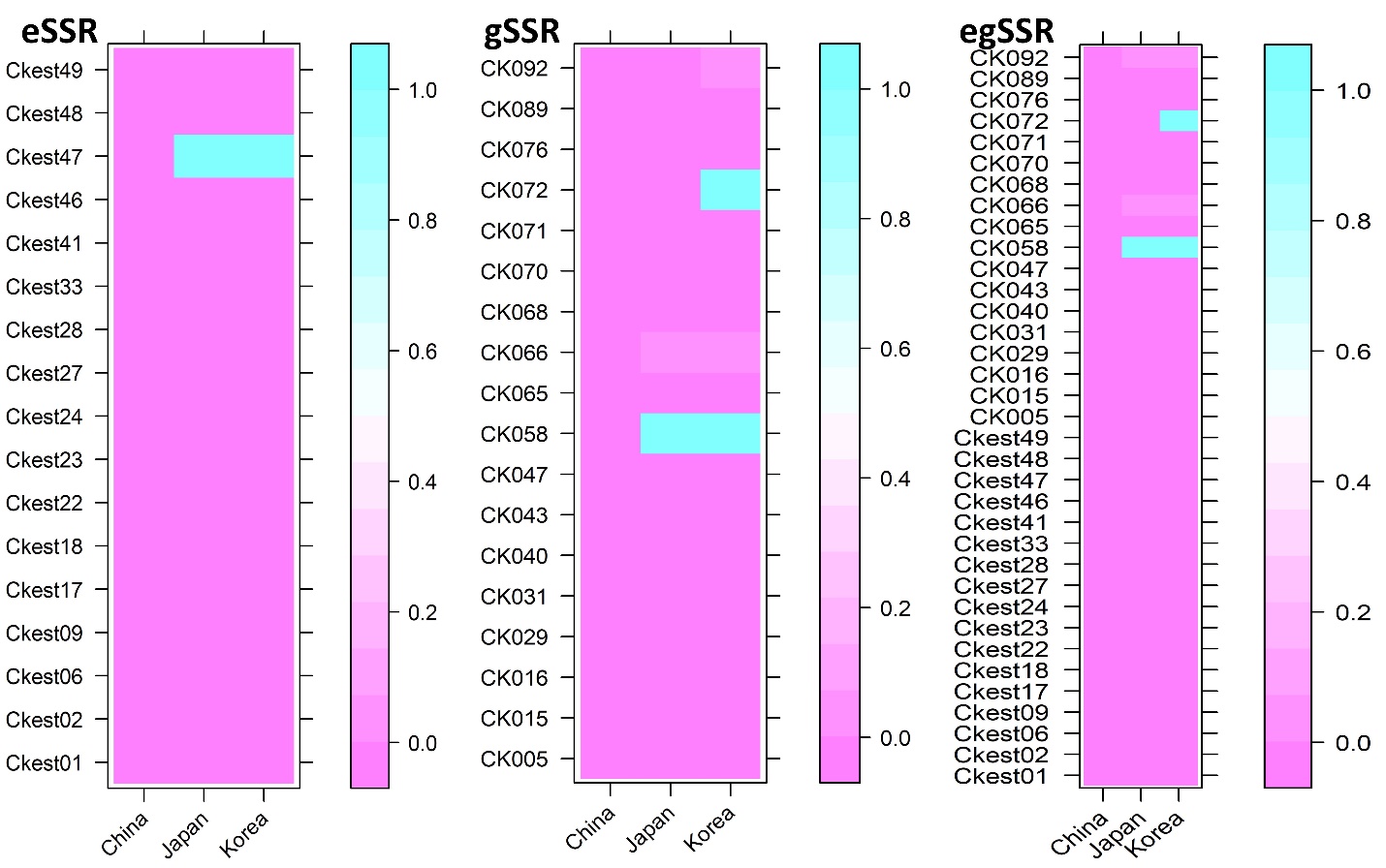


Figure C2. Hardy-Weinberg equilibrium (HWE) for the *Cornus kousa* combined dataset (egSSR; left panel); genic short sequence repeat markers SSRs (eSSR; middle panel); and genomic SSRs (gSSR; right panel). For each dataset, the respective loci (vertical Y axis) and populations (horizontal X axis) are indicated. Lower values (pink) indicate violations of the HWE assumptions, based on 1,000 permutations of each dataset, respectively.


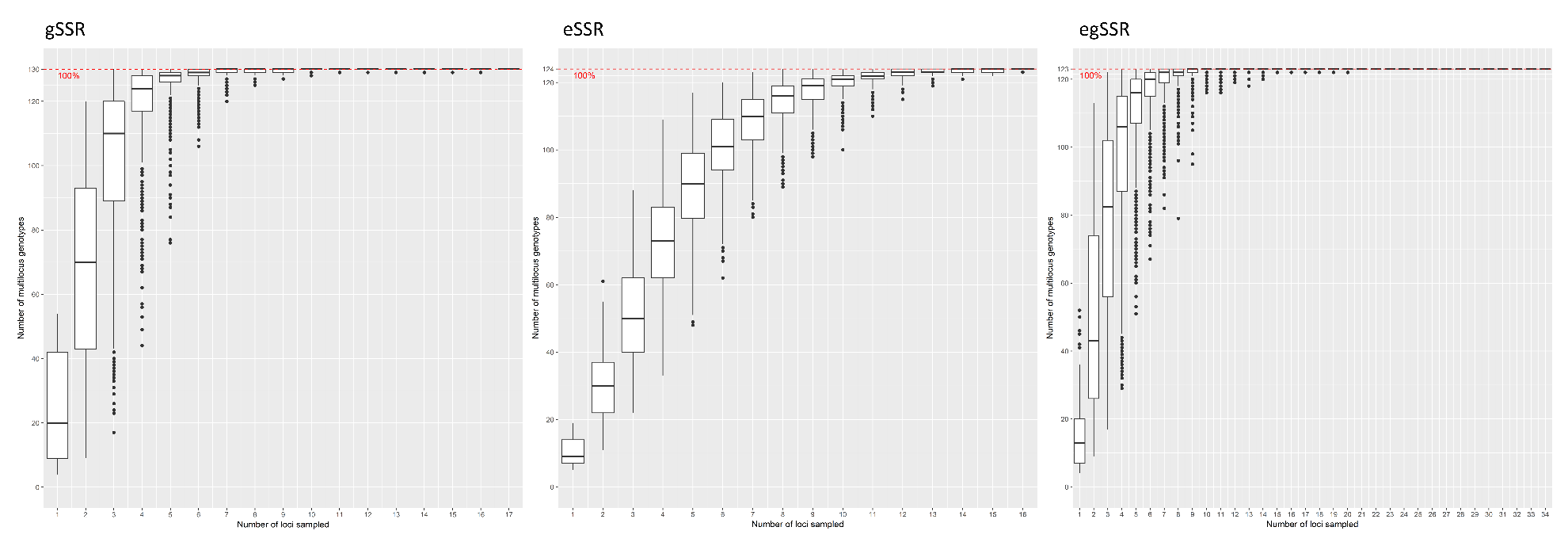
Figure C3. Genotype accumulation curves (GACs) for the *Cornus kousa* genomic short sequence repeat markers (gSSR; left panel), genic SSRs (eSSR; middle panel), and combined dataset (egSSR; right panel). Each graph indicates number of loci necessary to saturate the Multi-Locus Genotypes (MLGs) detected in each dataset, respectively.


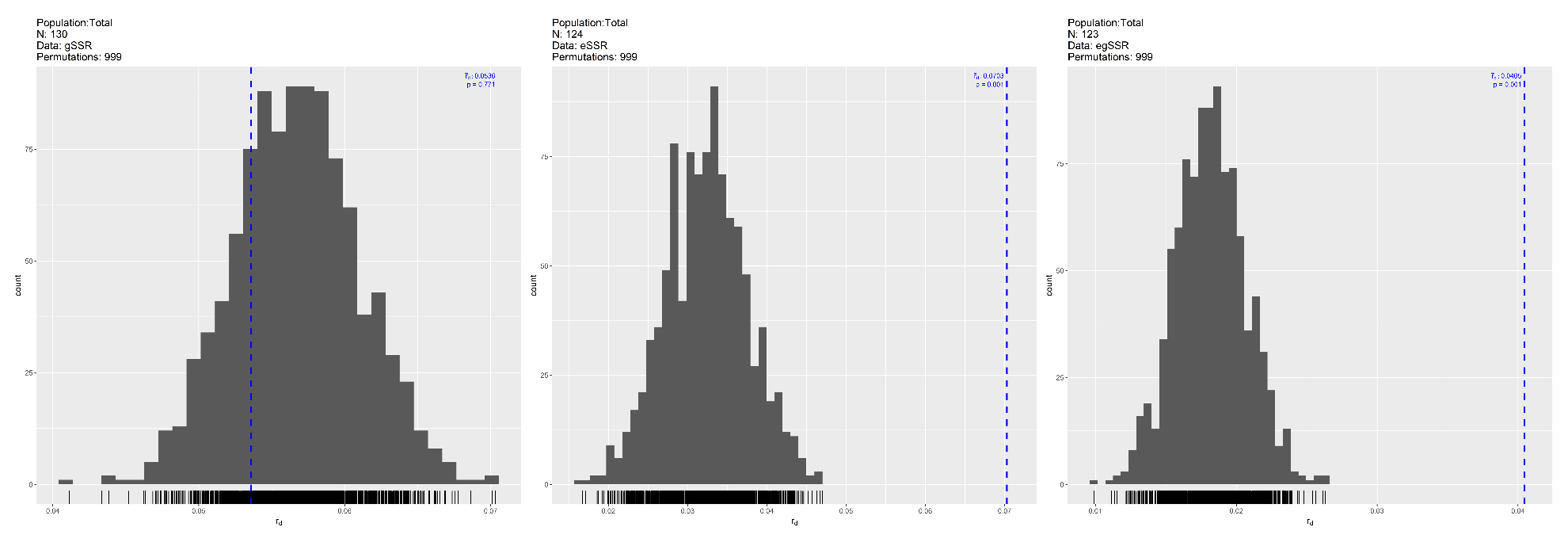
Figure C4. Assessment of clonality by Index of Association for the *Cornus kousa* genomic short sequence repeat markers (gSSR; left panel), genic SSRs (eSSR; middle panel), and combined dataset (egSSR; right panel). Each graph indicates the standardized overall index of association$(\bar{r}$_d_) with the corresponding statistical support (p), respectively.


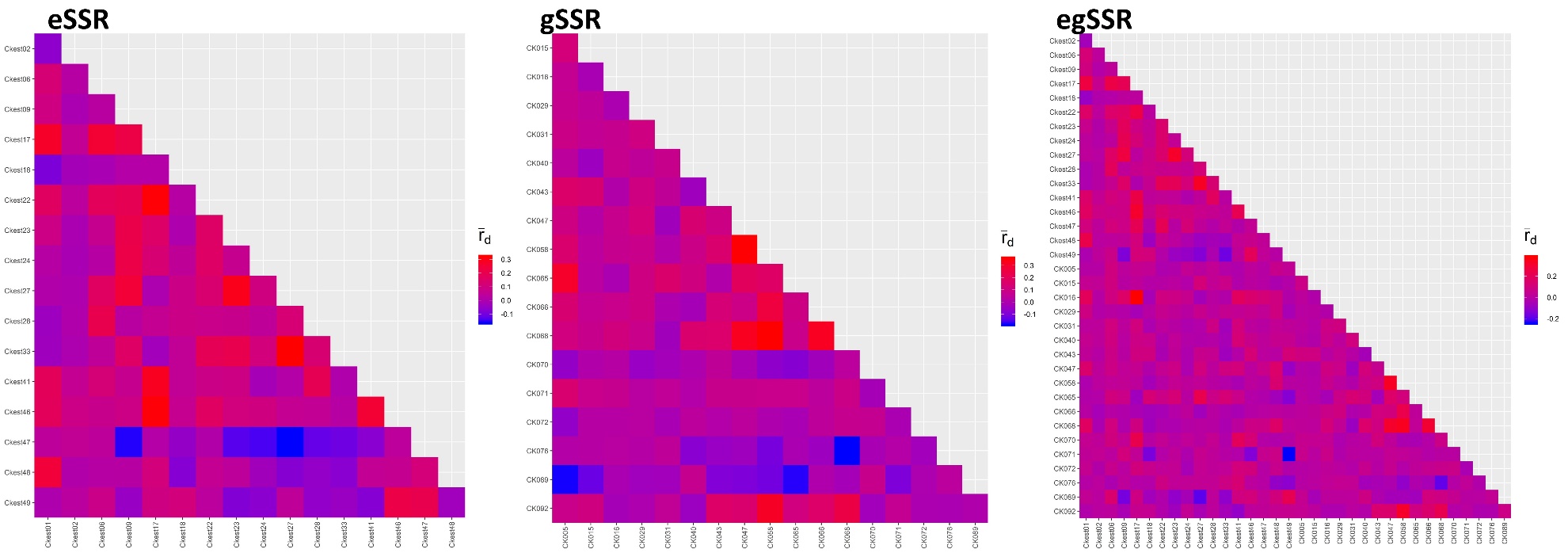


Figure C5. Assessment of Linkage Disequilibrium by standardized overall Index of Association $(\bar{r}$_d_) for the *Cornus kousa* genomic short sequence repeat markers (gSSR; left panel), genic SSRs (eSSR; middle panel), and combined dataset (egSSR; right panel). Each graph indicates the pairwise standardized overall index of association among the loci (indicated at the axes), with the corresponding scales, respectively. Higher values (red) denote pairs of loci more likely to be inherited together (linked), and lower values (negative; blue) denote pairs of loci more likely to not be inherited together (unlinked).


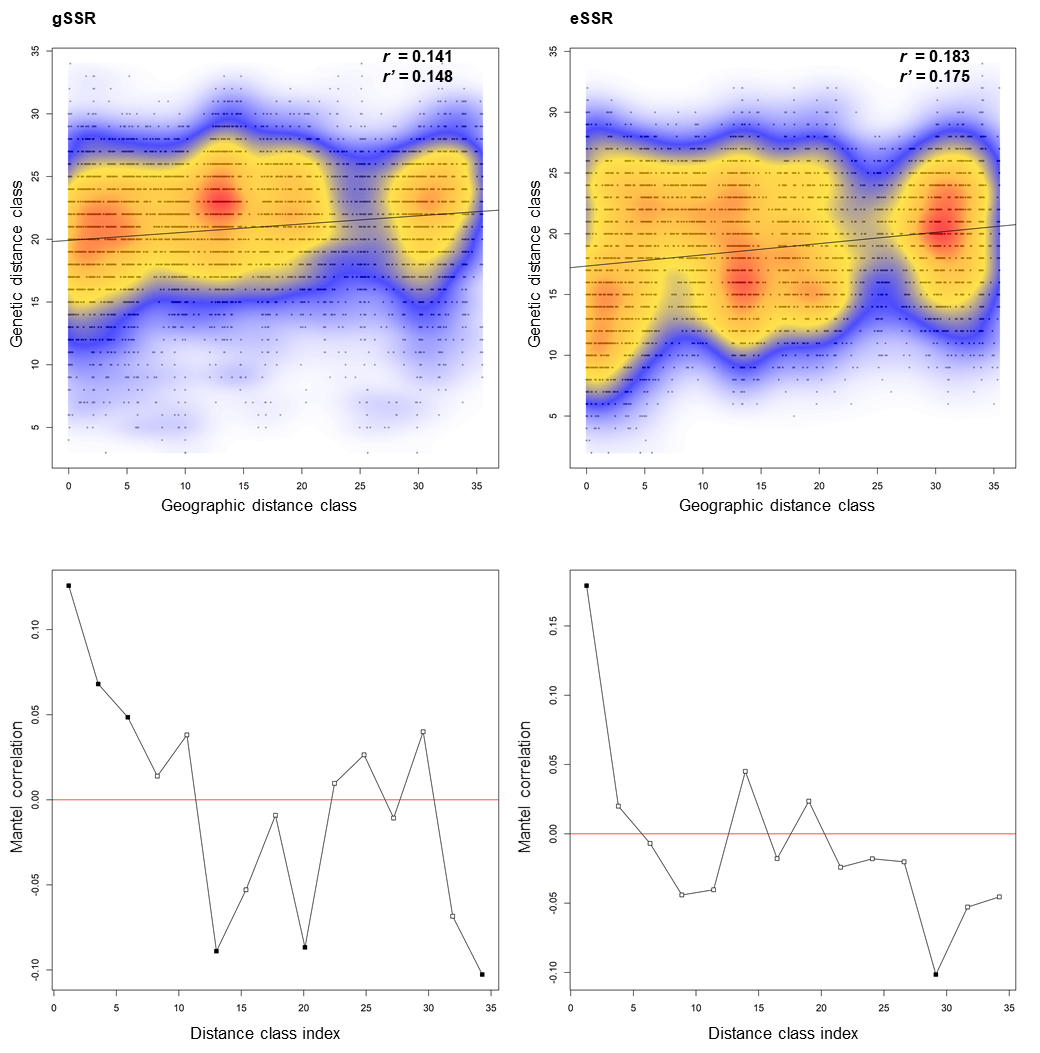

Figure C6. Mantel (top) and Mantel correlogram (bottom) tests for the isolation-by-distance analyses of the *Cornus kousa* eSSR and gSSR datasets (labeled on top). Data from genotyping the gDNA collection were investigated for the correlation between geographic and genetic distances using packages MASS, ade4, and vegan in R with 1,000 permutations. Correlograms marked with solid symbols are significant at *P* < 0.001.


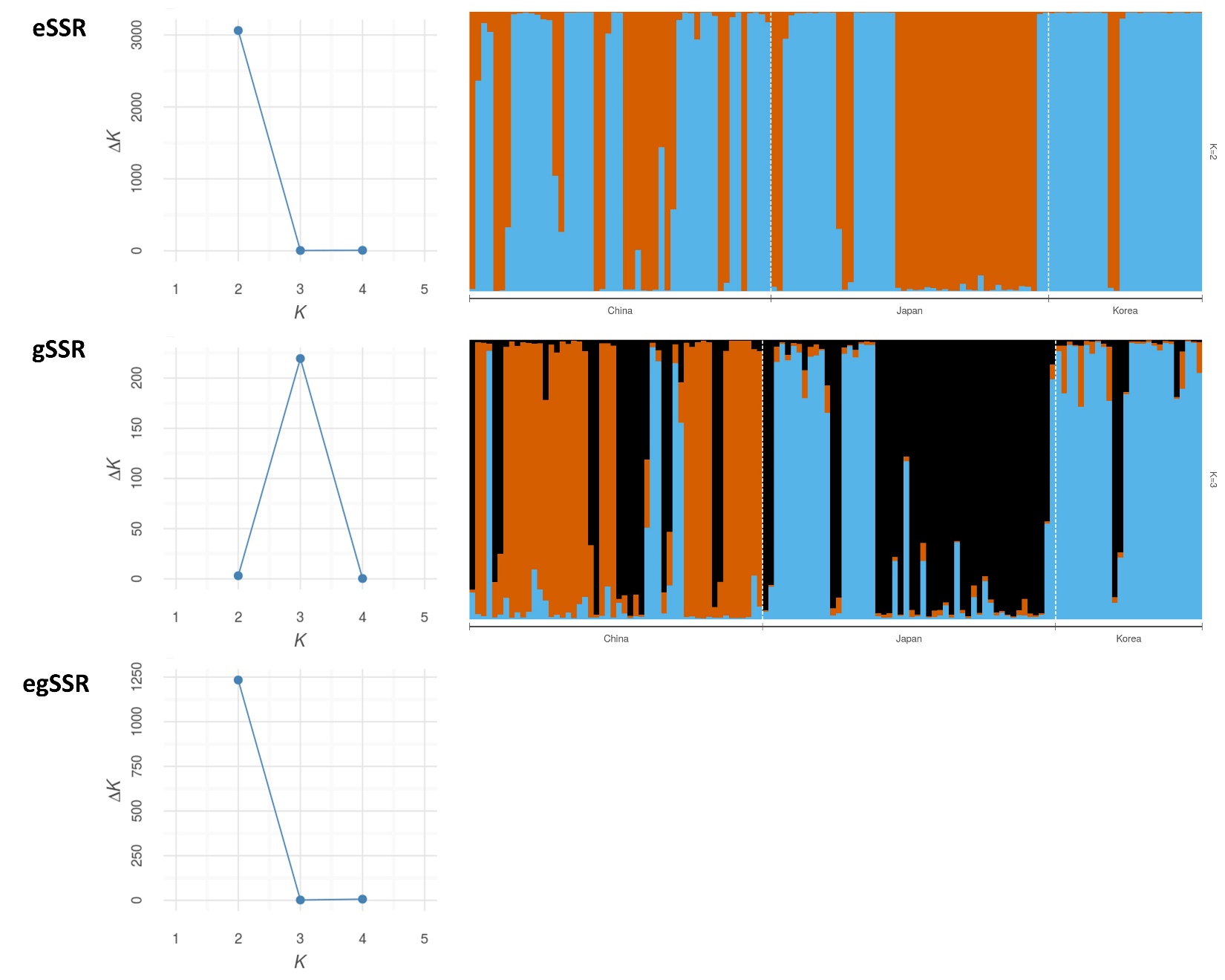


Figure C7. Bayesian clustering of the *Cornus kousa* datasets using Structure and Evanno method. Presented are the results for the eSSR and gSSR genotyped datasets. Structure was run for K = 1 through 5, using 30 independent Markov chains, with burn-in of 250,000 and runs of 750,000 steps. Presented are the Bayesian probabilities results for K = 2 and 3, depending on the analyses of the Structure results using the Evanno’s method with Structure harvester (Evanno, Regnaut, and Goudet 2005; Earl and Vonholdt 2012) to determine the optimal number of clusters (grapsh on the left-hand side). PopHelper was used to merge the runs of the 30 output files (Francis 2017).


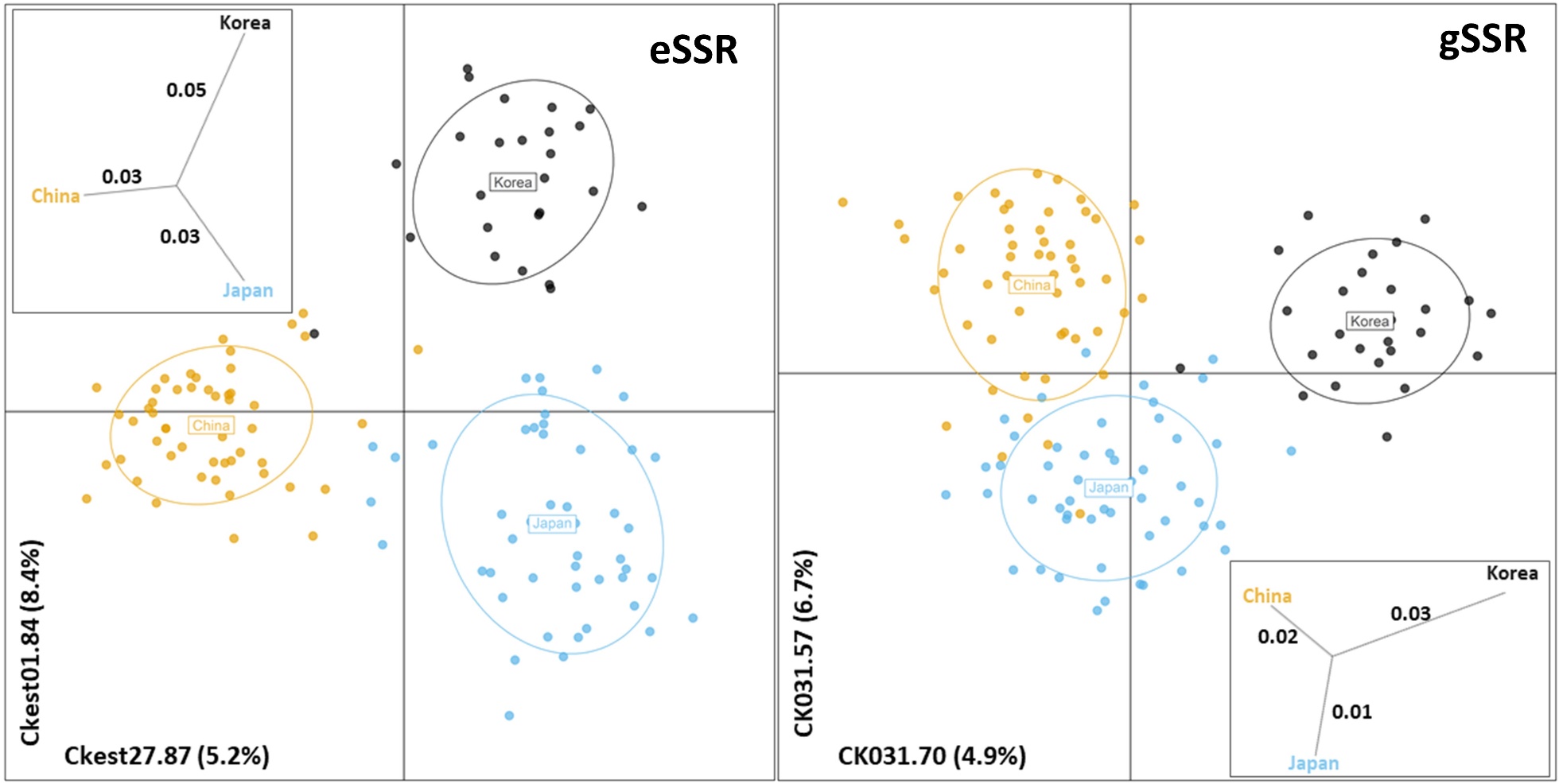
Figure C8. Discriminant Analyses of the Principal Components (DAPC) of the *Cornus kousa* datasets, genotyped using the genic SSRs (eSSR; left) or the genomic SSRs (gSSRs; right). Data from each dataset were investigated for their respective molecular variance partitioning using packages ade4 and adegenet in R/RStudio with the number of retained Principal Components (PCA eigenvalues) optimized and cross-checked over 1,000 permutations of each dataset, respectively. For each plot, the alleles explaining the most variance are indicated at respective axes, with their % contributions in parenthesis. Inserts: Unrooted neighbor-joining tree of pairwise genetic distances (F*_ST_*, Nei 1978) among the sub-populations of *C. kousa* analyzed in this study.


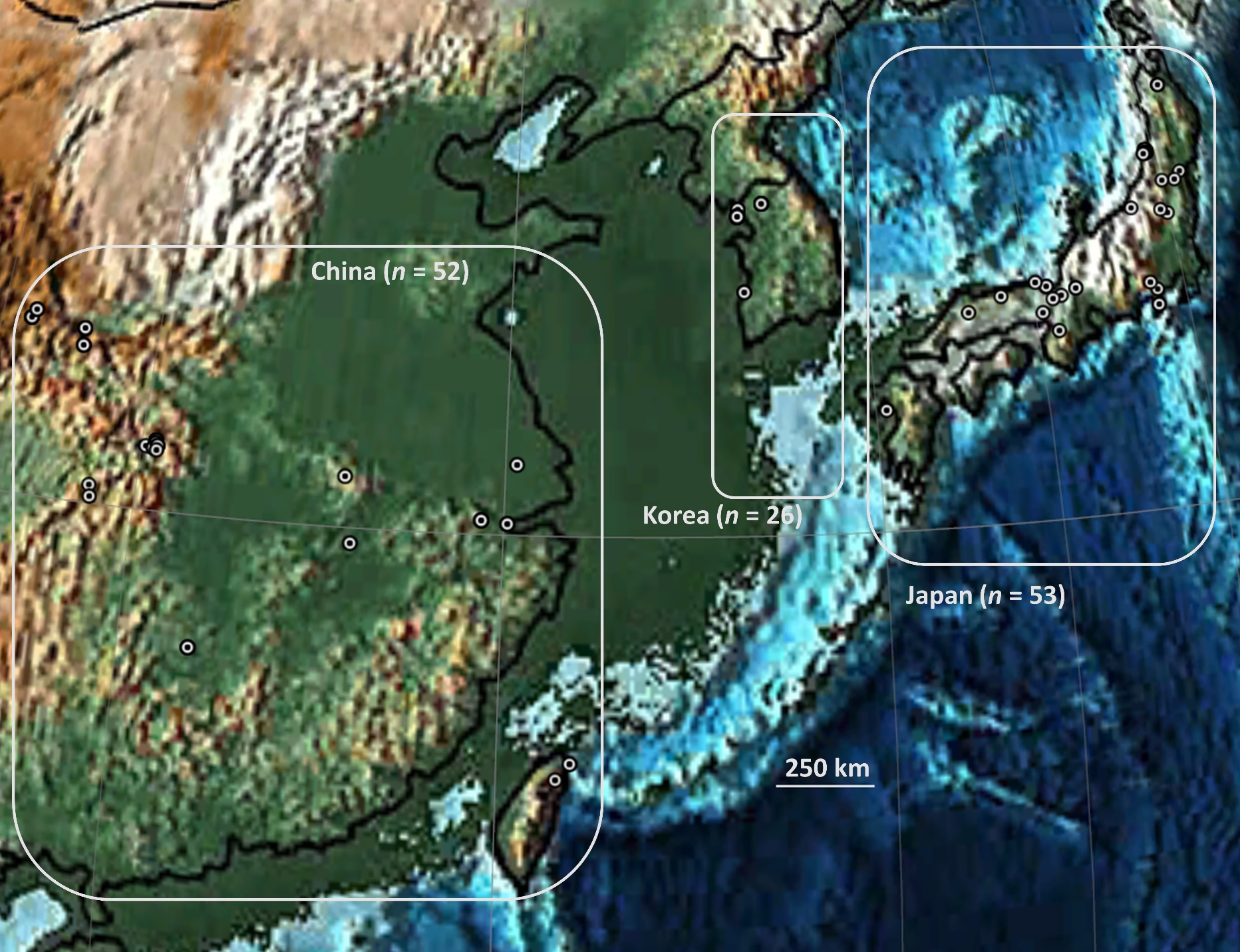
Figure C9. Map of the origin of locations of the *Cornus kousa* individuals sampled for the study. The GPS locations were visualized at the Last Glacial Maximum map (Pleistocene, 21,000 years ago; Scotese 2014). Eye altitude: 4,010 km above sea level. Present-day national borders are marked in black contours. Each sub-population is marked with the country of origin, and their respective counts are listed out. Individuals overlap in several locations due to the scale of the presentation. Also see Table 1 and Supplementary File SF1. The region maps were visualized using Google Earth Pro version 7.3.2.5491. The present-time GPS coordinates of our *C. kousa* collection are shown as whitish hollow circles. The scale line indicates a ground level distance of 250 km.

**Literature cited:**

Amos, W., Hoffman, J. I., Frodsham ,A., Zhang, L. , Best, S. & Hill, A. V. S. (2007). Automated binning of microsatellite alleles: problems and solutions. *Molecular Ecology Notes* **7** (1):10-14.

Atkinson, B. A. (2018). The critical role of fossils in inferring deep‐node phylogenetic relationships and macroevolutionary patterns in Cornales. *American Journal of Botany* **105** (8):1401-1411.

Bai, C. K., Alverson W. S., Follansbee, A., & Waller D. M. (2012). New reports of nuclear DNA content for 407 vascular plant taxa from the United States. *Annals of Botany* **110** (8):1623-1629.

Bhargava, A. & Fuentes, F. F. (2010). Mutational dynamics of microsatellites. *Molecular Biotechnology* **44** (3):250-266.

Bird, C. E., Karl, S. A., Smouse, P. E., & Toonen, R. J. (2011). Detecting and measuring genetic differentiation. *Phylogeography and Population Genetics in Crustacea* **19** (3):31-55.

Call, A. B. (2012). Phylogeography and species distribution modeling of the flowering dogwood, *Cornus florida* L. (Cornaceae). MSc, Plant Biology, North Carolina State University, Raleigh, North Carolina, USA.

Call, A., Sun, Y.-X., Yu, Y., Pearman, P. B., Thomas, D. T., Trigiano, R. N., Carbone, I., & Xiang, Q.-Y. (2015). Genetic structure and post‐glacial expansion of *Cornus florida* L. (Cornaceae): integrative evidence from phylogeography, population demographic history, and species distribution modeling. *Journal of Systematics and Evolution* **54** (2):136-151.

Cappiello, P., & Shadow, D. (2005). *Dogwoods: The Genus Cornus*. Timber Press.

Cornuet, J.-M., Pudlo, P., Veyssier, J., Dehne-Garcia, A., Gautier, M., Leblois, R., Marin, J.-M., & Estoup, A. (2014). DIYABC v2. 0: a software to make approximate Bayesian computation inferences about population history using single nucleotide polymorphism, DNA sequence and microsatellite data. *Bioinformatics* **30** (8):1187-1189.

Cornuet, J.-M., Ravigné, V., & Estoup, A. (2010). Inference on population history and model checking using DNA sequence and microsatellite data with the software DIYABC (v1. 0). *BMC Bioinformatics* **11** (1):401.

Cornuet, J.-M., & Luikart, G. (1996). Description and power analysis of two tests for detecting recent population bottlenecks from allele frequency data. *Genetics* **144** (4):2001-2014.

Dermen, H. (1932). Cytological studies of *Cornus*. *Journal of the Arnold Arboretum* **13** (4):410-416.

Dray, S, &Dufour, A, B. (20017). The ade4 package: implementing the duality diagram for ecologists. *Journal of Statistical Software* **22** (4):1-20.

Ellis, J. R. & Burke, J. M. (2007). EST-SSRs as a resource for population genetic analyses. *Heredity* **99** (2):125.

Evanno, G., Regnaut, S., & Goudet, J. (2005). Detecting the number of clusters of individuals using the software STRUCTURE: a simulation study. *Molecular Ecology* **14** (8):2611-2620.

Francis, R. M. (2017). pophelper: an R package and web app to analyse and visualize population structure. *Molecular Ecology Resources* **17** (1): 27-32.

Gibbons, L. G., Janson, E. M., Hittinger, C. T., Johnston, M., Abbot, P., & Rokas, A. (2009). Benchmarking next-generation transcriptome sequencing for functional and evolutionary genomics. *Molecular Biology and Evolution* **26** (12):2731-2744.

Goldblatt, P. (1978). A contribution to cytology in *Cornales*. *Annals of the Missouri Botanical Garden* 650-655.

Goudet, J., Raymond, M., de Meeus, T., & Rousset, F. (1996). Testing differentiation in diploid populations. *Genetics* **144** (4):1933-40.

Goudet, J. (2005). Hierfstat, a package for R to compute and test hierarchical F‐statistics. *Molecular Ecology Resources* **5** (1):184-186.

Hadziabdic, D. (2010). Evaluation of genetic diversity of flowering dogwood (*Cornus florida* L.) in the eastern United States using microsatellites. PhD diss., University of Tennessee – Knoxville.

Hadziabdic, D., Fitzpatrick, B. M., Wang, X., Wadl, P. A., Rinehart, T. A., Ownley, B. H., Windham, M. T., & Trigiano, R. N. (2010). Analysis of genetic diversity in flowering dogwood natural stands using microsatellites: the effects of dogwood anthracnose. *Genetica* **138** (9-10):1047-1057.

Hadziabdic, D., Wang, X., Wadl, P. A., Rinehart, T. A., Ownley, B. H., & Trigiano, R. N. (2012). Genetic diversity of flowering dogwood in the Great Smoky Mountains National Park. *Tree Genetics & Genomes* **8** (4):855-871.

Hagan, A. K., Hardin, B., Gilliam, C. H., Keever, G. J., Williams, J. D., & Eakes, J. (1998). Susceptibility of cultivars of several dogwood taxa to powdery mildew and spot anthracnose. *Journal of Environmental Horticulture* **16** (3):147-151.

Hardy, O. & Vekemans, X. (2015). SPAGeDi 1.5. A program for Spatial Pattern Analysis of Genetic Diversity. User’s manual http://ebe. ulb. ac. be/ebe/SPAGeDi_files/SPAGeDi_1. 5_Manual. pdf. Université Libre de Bruxelles, Brussells, Belgium.

Hey, J., Chung, Y., Sethuraman, A., Lachance, J., Tishkoff, S., Sousa, V. C., & Wang, Y. (2018). Phylogeny estimation by integration over isolation with migration models. *Molecular Biology and Evolution*, **35**(11): 2805-2818.

Jombart, T. (2008). adegenet: a R package for the multivariate analysis of genetic markers. *Bioinformatics* **24** (11):1403-5.

Jombart, T., Kamvar, Z. N., Collins, C., Lustrik, R., Beugin, M.-P., Knaus, B. J., & Jombart, M. T. (2018). Package ‘adegenet’.

Jost, L. (2008). G*_ST_* and its relatives do not measure differentiation. Molecular Ecology 17, 4015-4026.

Jost, L., Archer, F., Flanagan, S., Gaggiotti, O., Hoban, S., & Latch, E. (2018). Differentiation measures for conservation genetics. *Evolutionary applications*, 11(7):1139-1148.

Kamvar, Z. N., Brooks, J. C., & Grunwald, N. J. (2015). Novel R tools for analysis of genome-wide population genetic data with emphasis on clonality. *Frontiers in Genetics* **6**:208.

Kamvar, Z. N., Tabima, J. F., & Grünwald, N. J. (2014). Poppr: an R package for genetic analysis of populations with clonal, partially clonal, and/or sexual reproduction. *PeerJ* **2**:e281.

Kang, K.-S. & Lee, K.-Y. (2008). Trade-off between gene diversity and seedling production for gene conservation: *Cornus kousa* as a model. *Forest Ecology and Management* **255** (5-6):1446-1451.

Keir, K. R. 2008. Beautiful but lacking diversity: population genetics of Pacific Dogwood (*Cornus nuttallii* Audobon ex Torr. & A. Gray), University of British Columbia.

Keir, K. R., Bemmels, J. B., & Aitken, S. N. (2011). Low genetic diversity, moderate local adaptation, and phylogeographic insights in *Cornus nuttallii* (Cornaceae). *American Journal of Botany* 98 (8):1327-1336.

Kim, H.-J., Kwon, Y.-H., Park, K.-W., Oh, S.-H., & Choi, K. (2006). Use of ISSR marker for the variant identification in *Cornus kousa* Buerg. *Korean Journal of Plant Resources* **19** (4):509-514.

Kim, J., Ni, G., Kim, T., Chun, J.-Y., Kern, E. M. A., & Park, J.-K. (2019). Phylogeography of the highly invasive sugar beet nematode, *Heterodera schachtii* (Schmidt, 1871), based on microsatellites. *Evolutionary Applications* **12** (2):324-336.

Li, Y. H,, Windham, M. T., Trigiano, R. N., Fare, D., C., Spiers, J. M., & Copes, W. E. (2007). Evaluation for resistance to powdery mildew in *Cornus* species and hybrids using a leaf disk assay. *Journal of Environmental Horticulture* **25** (3):131-133.

Love, A. (1982). IOPB chromosome number reports LXXV. *Taxon* **31** (2):342-368.

Mattera, R. (2016). The Rutgers hybrid dogwood: naming and genetic diversity analysis. MSc, Plant Biology and Pathology, Rutgers University-Graduate School-New Brunswick, New Brunswick, New Jersey.

Mattera, R., Molnar, T., & Struwe, L. (2015). *Cornus* × *elwinortonii* and *Cornus* × *rutgersensis* (Cornaceae), new names for two artificially produced hybrids of big-bracted dogwoods. *PhytoKeys* **55**:93-111.

Merkle, S. A., Andrade, G. M., Nairn, C. J., Powell, W. A., & Maynard, C. A. (2007). Restoration of threatened species: a noble cause for transgenic trees. *Tree Genetics & Genomes* **3** (2):111-118.

Michalakis, Y & Excoffier, L. 1996. A generic estimation of population subdivision using distances between alleles with special reference for microsatellite loci. *Genetics* 142, 1061-1064.

Molnar, T. J, Muehlbauer, M., Wadl, P. A., & Capik, J. M. (2017). ‘Rutpink’(Scarlet Fire^®^) kousa dogwood. *HortScience* **52** (10):1438-1442.

Morgulis, A., Gertz, E. M., Schaffer, A. A., & Agarwala, R. (2006). A fast and symmetric DUST implementation to mask low-complexity DNA sequences. *Journal of Computational Biology* **13** (5):1028-40.

Nei, M.(1972). Genetic distance between populations. *The American Naturalist* **106** (949):283-292.

Nielsen, R., Tarpy, D. R., & Reeve, H. K. (2003). Estimating effective paternity number in social insects and the effective number of alleles in a population. *Molecular Ecology*, **12**(11), 3157-3164.

Nowicki, M., Boggess, S. L., Saxton, A. M., Hadziabdic, D., Xiang, Q. J., Molnar, T., Huff, M. L., Staton, M. E., Zhao, Y. & Trigiano, R. N. (2018). Haplotyping of *Cornus florida* and *C. kousa* chloroplasts: Insights into species-level differences and patterns of plastic DNA variation in cultivars. *PLoS One* **13** (10):e0205407.

Nowicki, M., Schilling, E. E., Boggess, S. L., Houston, L. C., Huff, M. L., Staton, M. E., Lampley, J. A., & Trigiano, R. N. (2019). Development and characterization of genic microsatellites for the ornamental plant Green and Gold (*Chrysogonum virginianum*). *HortScience* **54** (2):395-400.

Nowicki, M., Zhao, Y., Boggess, S. L., Fluess, H., Payá-Milans, M., Staton, M. E., Houston, L. C., Hadziabdic, D., & Trigiano, R. N. (2019). *Taraxacum kok-saghyz* (rubber dandelion) genomic microsatellite loci reveal modest genetic diversity and cross-amplify broadly to related species. *Scientific Reports* **9** (1):1915.

Oksanen, J., Blanchet, F. G., Kindt, R., Legendre, P., Minchin, P. R., O’hara, R. B., Simpson, G. L., Solymos, P., Stevens, M. H., Wagner, H., &Oksanen. M. J. (2013). Package ‘vegan’. Community ecology package, version 2.5-4. 2013 Dec 12;2(9).

Pfarr, E., Vaiciunas, J., Kubik, C., Capik, J. M., Honig, J., & Molnar, T. (2018). Preliminary results of a genotyping-by-sequencing diversity study of big-bracted dogwood (*Cornus* spp.) cultivars. *HortScience* **53** (9).

Piry, S., Luikart, G., & Cornuet, J. M. (1999). BOTTLENECK: a computer program for detecting recent reductions in the effective population size using allele frequency data. *Journal of Heredity* **90**:502-503.

Prevosti, A. (1974). La distancia genética entre poblaciones. *Miscellanea Alcobe* **68**:109-118.

Pritchard, J. K., Stephens, M., & Donnelly, P. (2000). Inference of population structure using multilocus genotype data. *Genetics* **155** (2):945-959.

R Core Team. (2017). R: A language and environment for statistical computing. Paper read at R Foundation for Statistical Computing; 2017, at Vienna, Austria.

Raquin, A.-L., Depaulis, F., Lambert, A., Galic, N., Brabant, P., & Goldringer, I. (2008). Experimental estimation of mutation rates in a wheat population with a gene genealogy approach. *Genetics* **179** (4):2195-2211.

Riser, J. P., Emel, S. L., & Roalson, E. H. (2019). Genetics and ecological niche define species boundaries in the dwarf milkweed clade (*Asclepias*: Asclepiadoideae: Apocynaceae). *International Journal of Plant Sciences* **180** (2):160-177.

Rubinsztein, D. C., Amos, W., Leggo, J., Goodburn, S., Jain, S., Li, S.-H., Margolis, R. L., Ross, C. A., & Ferguson-Smith M. A. (1995). Microsatellite evolution—evidence for directionality and variation in rate between species. *Nature Genetics* **10** (3):337.

Schmidt, J. F. (1995). *Cornus kousa* (dogwood) tree: Schmred: US Patent PP9,283.

Scotese, C.R., 2014. Atlas of Neogene Paleogeographic Maps (Mollweide Projection), Maps 1-14, Volume 1, The Cenozoic, PALEOMAP Atlas for ArcGIS, PALEOMAP Project, Evanston, IL.

Seeram, N. P, Schutzki, R., Chandra, A., & Nair, M. G. (2002). Characterization, quantification, and bioactivities of anthocyanins in *Cornus* species. *Journal of Agricultural and Food Chemistry* **50** (9):2519-2523.

Shannon, C. E. (2001). A mathematical theory of communication. *ACM SIGMOBILE Mobile Computing and Communications Review* **5**:3–55.

Shearer, K. & Ranney, T. G. (2013). Cytometric and cytological analyses of cultivated dogwoods (*Cornus* spp.). Paper read at Southern Nursery Association Research Conference, at Atlanta, GA.

———. (2013). Ploidy levels and relative genome sizes of species, hybrids, and cultivars of dogwood (*Cornus* spp.). *HortScience* **48** (7):825-830.

Simao, F. A., Waterhouse, R. M., Ioannidis,P., Kriventseva, E. V., & Zdobnov, E. M. (2015). BUSCO: assessing genome assembly and annotation completeness with single-copy orthologs. *Bioinformatics* **31** (19):3210-3212.

Simpson, E. H. (1949) Measurement of diversity. *Nature* **163**: 688, 1949 doi:10.1038/163688a0

Slatkin, M. (1995). A measure of population subdivision based on microsatellite allele frequencies. *Genetics* **139** (1): 457-462.Song, J. H., Goo, Y. B., Han, S. H., Yang, B. H., & Park, H. S. (2006). The variation of germination, growth and leaf form of open-pollinated progenies of *Cornus kousa* Buerger ex Miquel in Korea. *Journal of Korean Forestry Society* **95** (3): 261-261.

Stoddart JA, Taylor JF. 1988. Genotypic diversity: estimation and prediction in samples. *Genetics* **118**:705-711

Thurn, M., Lamb, E., & Eshenaur, B. (2018). *Cornus*, edited by C. University: New York State Integrated Pest Management Program.

Trigiano, R. N., Ament, M. H., Windham, M. T., & Moulton, J. K. (2004). Genetic profiling of red-bracted *Cornus kousa* cultivars indicates significant cultivar synonomy. *HortScience* **39** (3):489-492.

Untergasser, A., Cutcutache, I., Koressaar, T., Ye, J., Faircloth, B. C., Remm, M., & Rozen, S. G. (2012). Primer3—new capabilities and interfaces. *Nucleic Acids Research* **40** (15):e115-e115.

USDA-NASS. *Census of horticultural specialties (2012), Table 21: Deciduous flowering trees* 2014 [cited 2019-04-15. Available from https://www.agcensus.usda.gov/Publications/2012/Online_Resources/Census_of_Horticulture_Specialties/.

Vareed, S. K, Reddy, M. K., Schutzki, R E., & Nair, M. G. (2006). Anthocyanins in *Cornus alternifolia*, *Cornus controversa*, *Cornus kousa* and *Cornus florida* fruits with health benefits. *Life Sciences* **78** (7):777-784.

Venables, V. N., & Ripley, B. D. (2002). *Modern Applied Statistics with S.* New York, NY, USA: Springer Science+ Business Media.

Wadl, P. A., Skinner, J. A., Dunlap, J. R., Reed, S. M., Rinehart, T. A., Pantalone, V. R., & Trigiano, R. N. (2009). Honeybee-mediated controlled pollinations in *Cornus florida* and *C. kousa* intra- and interspecific crosses. *HortScience* **44** (6):1527-1533.

Wadl, P. A., Wang, X., Scheffler, B. E., Rinehart, T. A., & Trigiano, R N. (2008). Microsatellites from kousa dogwood (*Cornus kousa*). *Molecular Ecology Resources* **8** (4):780-2.

Wadl, P. A., Wang, X. W., Moulton, J K., Hokanson, S. C., Skinner, J. A., Rinehart, T. A., Reed, S. M., Pantalone, V. R., & Trigiano, R. N. (2010). Transfer of *Cornus florida* and *C. kousa* simple sequence repeats to selected *Cornus* (Cornaceae) species. *Journal of the American Society for Horticultural Science* **135** (3):279-288.

Wadl, P. A., X. W. Wang, A. N. Trigiano, J. A. Skinner, M. T. Windham, R. N. Trigiano, T. A. Rinehart, S. M. Reed, & V. R. Pantalone. (2008). Molecular identification keys for cultivars and lines of *Cornus florida* and *C. kousa* based on simple sequence repeat loci. *Journal of the American Society for Horticultural Science* 133 (6):783-793.

Wadl, P.A., Saxton, A.M., Wang, X., Pantalone, V.R., Rinehart, T.A. & Trigiano, R.N. (2011). Quantitative trait loci associated with red foliage in *Cornus florida* L. *Molecular breeding*, **27**(3):409-416.

Wadl, P. A, Szyp-Borowska, I., Piórecki, N., Schlarbaum, S. E., Scheffler, B. E., & Trigiano, R. N. (2014). Development of microsatellites from *Cornus mas* L. (Cornaceae) and characterization of genetic diversity of cornelian cherries from China, central Europe, and the United States. *Scientia Horticulturae* **179**:314-320.

Wadl, P. A., Windham, M. T., Evans, R., & Trigiano, R. T. (2014). Three new cultivars of *Cornus kousa*: Empire, Pam’s Mountain Bouquet, and Red Steeple. *HortScience* **49** (9):1230-1233.

Wang, X., Wadl, P.A., Rinehart, T.A., Scheffler, B.E., Windham, M.T., Spiers, J.M., Johnson, D.H. & Trigiano, R.N. (2009). A linkage map for flowering dogwood (*Cornus florida* L.) based on microsatellite markers. *Euphytica*, **165**(1):165-175.

Yu, Y., Xiang, Q., Manos, P. S., Soltis, D. E., Soltis, P. S., Song, B.-H., Cheng, S., Liu, X., & Wong, G. (2017). Whole-genome duplication and molecular evolution in *Cornus* L.(Cornaceae)–Insights from transcriptome sequences. *PLoS One* **12** (2):e0171361.

Yuan, J.Q., Fang, Q., Liu, G.H. & Fu, X.X. (2019). Low divergence among natural populations of *Cornus kousa* subsp. *chinensis* revealed by ISSR markers. *Forests*, **10**(12), p.1082.

Zhang, J., Yao, J., Hu,Z.-M., Jueterbock, A., Yotsukura, N., Krupnova, T. N., Nagasato, C., & Duan, D. (2019). Phylogeographic diversification and postglacial range dynamics shed light on the conservation of the kelp *Saccharina japonica*. *Evolutionary Applications* **12** (4):791-803.
