# Supplementary material for: Species diversity and phylogeography of Cornus kousa (Asian dogwood) captured by genomic and genic microsatellites
Source: Ecol Evol. 2020 Jul 11;10(15):8299–312. doi: 10.1002/ece3.6537 (PMC7417245; doi:10.1002/ece3.6537)
Supplement: Supplementary file 4 — Table S1‐S3 [file ECE3-10-8299-s001.docx]

Supplementary Table ST1. AMOVAresults.

| **Source of variation** | **df** | **Sum of squares** | **Mean Sq** | **Sigma** | **% Variance** |
| --- | --- | --- | --- | --- | --- |
| **egSSR** | | | | | |
| **Between Populations** | 2 | 444.2579 | 222.1289 | 2.329866 | 9.27 |
| **Between Samples Within Populations** | 120 | 4525.155 | 37.70963 | 14.89896 | 59.26 |
| **Within Samples** | 123 | 973.1388 | 7.911698 | 7.911698 | 31.47 |
| **Total** | 245 | 5942.552 | 24.25531 | 25.14053 | 100 |
|  | **Φ_ST_** | **Φ_SP_** | **Φ_PT_** |  |  |
|  | 0.685301 | 0.653158 | 0.092674 |  |  |
| **eSSR** | | | | | |
| **Between Populations** | 2 | 217.8622 | 108.9311 | 1.154489 | 11.23 |
| **Between Samples Within Populations** | 121 | 2039.013 | 16.85134 | 7.724139 | 75.13 |
| **Within Samples** | 124 | 173.9801 | 1.403066 | 1.403066 | 13.64 |
| **Total** | 247 | 2430.855 | 9.841518 | 10.28169 | 100 |
|  | **Φ_ST_** | **Φ_SP_** | **Φ_PT_** |  |  |
|  | 0.863538 | 0.846277 | 0.112286 |  |  |
| **gSSR** | | | | | |
| **Between Populations** | 2 | 210.0277 | 105.0139 | 1.003112 | 6.64 |
| **Between Samples Within Populations** | 127 | 2737.475 | 21.55492 | 7.438841 | 49.20 |
| **Within Samples** | 130 | 868.0414 | 6.677241 | 6.677241 | 44.16 |
| **Total** | 259 | 3815.544 | 14.73183 | 15.11919 | 100 |
|  | **Φ_ST_** | **Φ_SP_** | **Φ_PT_** |  |  |
|  | 0.55836 | 0.526976 | 0.066347 |  |  |

Supplementary Table ST2. *R^2^* statistics and changes from the observed *R^2^* values when each *Cornus kousa* sub-population and inferred cluster were removed in turn.

| **Dataset** | **eCK** | | **gCK** | | **egCK** | |
| --- | --- | --- | --- | --- | --- | --- |
|  | **Average** | **St.Dev.** | **Average** | **St.Dev.** | **Average** | **St.Dev.** |
| Overall R^2^ for dataset^a^ | 0.170^***^ | 5.65E-17 | 0.412^***^ | 0.004 | 0.205^***^ | 0.005 |
|  |  |  |  |  |  |  |
| R^2^ without "Korea" | 0.040 | 1.41E-17 | 0.290 | 5.65E-17 | 0.080 | 2.82E-17 |
| R^2^ without "Japan" | 0.140 | 5.65E-17 | 0.330 | 0 | 0.130 | 8.47E-17 |
| R^2^ without "China" | 0.260 | 1.69E-16 | 0.420 | 0.002 | 0.300 | 0.002 |
|  |  |  |  |  |  |  |
| R^2^ without inferred BLUE^b^ | 0.170 | 5.65E-17 | 0.490 | 1.69E-16 | 0.205 | 0.005 |
| R^2^ without inferred ORANGE | 0.170 | 5.65E-17 | 0.410 | 1.13E-16 | 0.205 | 0.005 |
| R^2^ without inferred BLACK |  |  | 0.350 | 1.69E-16 |  |  |
| **Allele-freq. divergence among pops**^c^ | | | | | | |
|  | “China” | “Japan” | “China” | “Japan” | “China” | “Japan” |
| “Japan” | 0.142 |  | 0.196 |  | 0.347 |  |
| “Korea” | 0.113 | 0.185 | 0.131 | 0.183 | 0.350 | 0.344 |

^a^ ^***^ - significant at *P* < 0.0001

^b^ The color names (BLUE, ORANGE, BLACK) denote the clusters inferred with Structure (Fig. 3) for K = 3 (gCK) or 2 (eCK; egCK) across the 30 independent Markov chains.

^c^ Net nucleotide distance, computed using point estimates of P.

Supplementary Table ST3. Results for the DIYABC analyses of the *Cornus kousa* genotyping datasets.

| **gSSR** | | | | | | | | | |
| --- | --- | --- | --- | --- | --- | --- | --- | --- | --- |
| **Parameter** | **mean** | **median** | **mode** | **q025** | **q050** | **q250** | **q750** | **q950** | **q975** |
| **N_China_^a^** | 5.21E+3 | 5.13E+3 | 5.14E+3 | 1.45E+3 | 1.84E+3 | 3.61E+3 | 6.73E+3 | 8.97E+3 | 9.46E+3 |
| **N_Japan_** | 5.66E+3 | 5.65E+3 | 6.19E+3 | 1.70E+3 | 2.19E+3 | 4.13E+3 | 7.20E+3 | 9.18E+3 | 9.57E+3 |
| **N_Korea_** | 2.38E+3 | 1.95E+3 | 1.37E+3 | 3.78E+2 | 5.21E+2 | 1.18E+3 | 3.03E+3 | 6.07E+3 | 7.58E+3 |
| **N_Ancestral_** | 5.74E+3 | 5.74E+3 | 5.18E+3 | 1.39E+3 | 1.88E+3 | 3.93E+3 | 7.65E+3 | 9.45E+3 | 9.72E+3 |
| **t_1_** | 3.18E+2 | 2.59E+2 | 1.58E+2 | 5.92E+1 | 7.70E+1 | 1.62E+2 | 4.00E+2 | 7.07E+2 | 8.67E+2 |
| **µmic_1_** | 4.14E-4 | 3.75E-4 | 3.00E-4 | 1.83E-4 | 2.05E-4 | 2.90E-4 | 4.98E-4 | 7.73E-4 | 8.60E-4 |
| **pmic_1_** | 2.74E-1 | 2.84E-1 | 3.00E-1 | 1.86E-1 | 2.10E-1 | 2.63E-1 | 2.97E-1 | 3.00E-1 | 3.00E-1 |
| **snimic_1_** | 4.58E-7 | 1.01E-7 | 1.00E-8 | 1.07E-8 | 1.16E-8 | 2.79E-8 | 4.08E-7 | 2.26E-6 | 3.47E-6 |
| **eSSR** | | | | | | | | | |
| **Parameter** | **mean** | **median** | **mode** | **q025** | **q050** | **q250** | **q750** | **q950** | **q975** |
| **N_China_** | 6.34E+3 | 6.55E+3 | 7.23E+3 | 2.08E+3 | 2.71E+3 | 5.00E+3 | 7.81E+3 | 9.39E+3 | 9.70E+3 |
| **N_Japan_** | 4.51E+3 | 4.25E+3 | 3.60E+3 | 9.09E+2 | 1.25E+3 | 2.76E+3 | 5.99E+3 | 8.76E+3 | 9.34E+3 |
| **N_Korea_** | 1.94E+3 | 1.41E+3 | 7.88E+2 | 2.27E+2 | 3.09E+2 | 8.02E+2 | 2.40E+3 | 5.80E+3 | 7.47E+3 |
| **N_Ancestral_** | 5.05E+3 | 4.92E+3 | 3.50E+3 | 7.02E+2 | 1.10E+3 | 3.04E+3 | 7.02E+3 | 9.30E+3 | 9.65E+3 |
| **t_1_** | 4.52E+2 | 3.30E+2 | 1.80E+2 | 5.32E+1 | 7.44E+1 | 1.88E+2 | 5.53E+2 | 1.10E+3 | 1.50E+3 |
| **µmic_1_** | 1.61E-4 | 1.35E-4 | 1.02E-4 | 5.80E-5 | 6.52E-5 | 9.85E-5 | 1.89E-4 | 3.44E-4 | 4.42E-4 |
| **pmic_1_** | 2.85E-1 | 2.94E-1 | 3.00E-1 | 2.26E-1 | 2.44E-1 | 2.79E-1 | 3.00E-1 | 3.00E-1 | 3.00E-1 |
| **snimic_1_** | 3.69E-7 | 7.34E-8 | 1.00E-8 | 1.03E-8 | 1.11E-8 | 2.25E-8 | 3.03E-7 | 1.69E-6 | 2.86E-6 |
| **egSSR** | | | | | | | | | |
| **Parameter** | **mean** | **median** | **mode** | **q025** | **q050** | **q250** | **q750** | **q950** | **q975** |
| **N_China_** | 5.33E+3 | 5.33E+3 | 5.55E+3 | 1.54E+3 | 2.01E+3 | 3.85E+3 | 6.71E+3 | 8.88E+3 | 9.42E+3 |
| **N_Japan_** | 4.68E+3 | 4.46E+3 | 3.63E+3 | 1.16E+3 | 1.50E+3 | 3.05E+3 | 6.12E+3 | 8.71E+3 | 9.21E+3 |
| **N_Korea_** | 1.64E+3 | 1.20E+3 | 8.86E+2 | 2.23E+2 | 3.08E+2 | 7.31E+2 | 1.98E+3 | 4.77E+3 | 6.30E+3 |
| **N_Ancestral_** | 4.72E+3 | 4.44E+3 | 3.65E+3 | 8.03E+2 | 1.16E+3 | 2.82E+3 | 6.52E+3 | 9.01E+3 | 9.47E+3 |
| **t_1_** | 2.21E+2 | 1.72E+2 | 1.11E+2 | 3.88E+1 | 5.10E+1 | 1.08E+2 | 2.64E+2 | 4.82E+2 | 6.13E+2 |
| **µmic_1_** | 3.23E-4 | 2.89E-4 | 2.48E-4 | 1.50E-4 | 1.66E-4 | 2.27E-4 | 3.79E-4 | 6.07E-4 | 7.10E-4 |
| **pmic_1_** | 2.89E-1 | 2.96E-1 | 3.00E-1 | 2.42E-1 | 2.57E-1 | 2.84E-1 | 3.00E-1 | 3.00E-1 | 3.00E-1 |
| **snimic_1_** | 3.47E-7 | 8.22E-8 | 1.00E-8 | 1.05E-8 | 1.14E-8 | 2.53E-8 | 3.15E-7 | 1.56E-6 | 2.45E-6 |

^a^ Parameters for the overall most-reliable scenario are listed out for each dataset. N_X_ – effective size of the given sub-population; t_1_ – estimated time since split (in generations); µmic_1_ – overall mutation rate (in mutations per locus per generation); pmic_1_ - parameter of the geometric distribution (P); snimic_1_ – estimated single nucleotide insertions/deletions frequency (a stringency criterion).
